# Impacts of particle morphology and rotation on optical manipulation

**DOI:** 10.1038/s41377-026-02403-5

**Published:** 2026-07-13

**Authors:** Weicheng Yi, Yuzhi Shi, Hongfei Jiao, Chengxing Lai, Haiyang Huang, Xinhua Dai, Xiaoyun Gong, Hui Zhang, Qinghua Song, Zhanshan Wang, Zeyong Wei, C. T. Chan, Cheng-Wei Qiu, Xinbin Cheng

**Affiliations:** 1 Institute of Precision Optical Engineering, School of Physics Science and Engineering, Tongji University, Shanghai, 200092 China; 2 MOE Key Laboratory of Advanced Micro-Structured Materials, Shanghai, 200092 China; 3 Shanghai Institute of Intelligent Science and Technology, Tongji University, Shanghai, 200092 China; 4 Shanghai Frontiers Science Center of Digital Optics, Shanghai, 200092 China; 5 Technology Innovation Center of Mass Spectrometry for State Market Regulation, Center for Advanced Measurement Science, National Institute of Metrology, Beijing, 100029 China; 6 Tsinghua Shenzhen International Graduate School, Tsinghua University, Shenzhen, 518055 China; 7 Department of Physics, The Hong Kong University of Science and Technology, Clear Water Bay, Kowloon, Hong Kong, China; 8 Department of Electrical and Computer Engineering, National University of Singapore, Singapore, 117583 Singapore

**Keywords:** Optical manipulation and tweezers, Nanophotonics and plasmonics, Applied optics

## Abstract

It is well known that nonspherical particles rotate continuously in light beams, with their rotation directions determined by the light spin. Here, we reveal that particle morphology (e.g., size, shape, length, etc.) and rotation angle are vital in influencing light–matter interactions, thereby optical torques (OTs) and forces, especially in a paraxial or slightly defocused optical system. Positive and negative OTs emerge with distinct rotation angles in light fields. Their competition can also freeze the particle’s rotation, leading to the stable lateral drift effect. These extraordinary OTs arise from the nonuniformly distributed optical force vectors in the paraxial trapping. Experimentally, we observe positive and negative OTs, and bilateral drift (zero OT) of various particles (e.g., long and short cylinders, triangles, irregular shapes, etc.) under the influence of inverse optical forces and torques. Our works delve into a fascinating domain where morphologies and rotation angles of particles play essential roles in optical manipulation, enriching the fundamental understanding of optical forces and torques.

## Introduction

Optical tweezers trap and rotate particles via optical forces and torques, enabling numerous physical and biomedical applications^1–7^. Nonsphericity, and more commonly, morphology is a common characteristic widely observed in bioparticles (e.g., proteins and DNA) and synthesised particles^8–11^. Compared with spherical particles, nonspherical ones endow unique properties in a great diversity of fields, enabling numerous applications such as sensing and catalysis^12–15^. One distinct character of a nonspherical particle is that it can experience a significant optical torque in a light field, when the light possesses the spin angular momentum (SAM). It is well established that nonspherical particles can undergo continuous rotation in response to the helicity of the incident light, serving as valuable tools for applications such as microfluidic stirring, biological stretching and quantum measurements^8,16–22^.

**Fig. 1:**
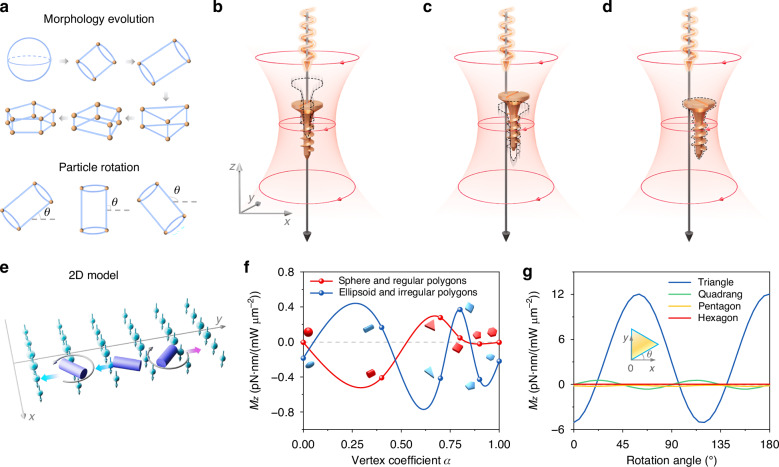
Morphology- and rotation-dependent optical torques. **a** Schematics of the morphologies (shape, size, length, etc.) and rotation of particles. **b** Schematics of a screw (representing a specific morphology of shape and size) rotating clockwise (top view) and moving downwards (−*y* direction) in a right-handed circular polarised (RCP, polarisation angle *φ* = 45°) beam by the positive OT. **c** In a paraxial system, when the rotation angle of the shaped particle changes, negative OT emerges, making the screw rotate counter-clockwise and move upwards (+*y* direction). **d** In a specific rotation angle, the screw may rotationally freeze under the balance of positive and negative OTs. **e** Illustration of reversible MRIOTs and optical forces in a 2D light field, which simplifies the 3D dynamics in optical trapping into a 2D model by converting the orbital degree into the lateral movement. This 2D model facilitates the investigation of those intriguing OTs and optical forces on a single plane. **f** Simulation of shape evolution to the OT. The sphere and ellipsoid can be evolved into various shapes by changing the vertex coefficient *α* (see Supplementary Materials). **g** Simulation of rotation-induced reversible OTs on various-shaped particles. Particle centres are placed at *x* = 200 nm. The side of the triangle is 1200 nm. All particles have the same areas. In (**f**, **g**), *w*_*x*_ = 1000 nm and *w*_*y*_ = 500 μm. OTs are calculated by normalising the intensity at (*x* = 0, *y* = 0) to 1 W μm^−2^

In a light field, a shaped particle with a distinctive morphology (Fig. 1a) can be treated as a dipole or multipole from the perspective of scattering theory, which can be trapped in a focused light beam and rotated with the SAM^23–26^. In most scenarios, the OT follows the direction of the SAM, called the positive OT^27–30^. Negative or left-handed OTs in the opposite direction of the SAM emerge with some special configurations, for instance, particle clusters^27,31–33^, chiral objects^34–36^, phase-gradient fields^28,29,37,38^, elliptical polarisations^24,39,40^, nonlinearity^41^, imaginary Poynting momentum^42^, vortex beams^43^, spin-gradient light field^44^, etc. In single-beam optical tweezers with normal incidence, a shaped particle on a focal plane can be trapped in the focal point. In this case, the particle can rotate continuously, with its orientation following the light spin (Fig. 1b). However, when the particle, particularly one of micro size, is trapped in a slightly defocused plane, the optical radiation pressure and gradient force lead to paraxial trapping. This off-axis trapping, though ubiquitously exhibited, is somehow ignored in the optical manipulation community. We hereby show that in this regime, the morphology (e.g., shape, length and size) and rotation of particles play essential roles in influencing the OT and optical force.

The negative OT mechanism introduced in this work is fundamentally distinct from all previously reported scenarios. For instance, in particle clusters, negative OTs arise from near-field coupling and collective interactions; in chiral objects, they originate from intrinsic chirality and spin angular momentum coupling; and in phase-gradient fields or nonlinear systems, they stem from structured light carrying orbital angular momentum (OAM) or nonlinear optical responses. Likewise, the present mechanism differs from those involving imaginary Poynting momentum, where electromagnetic field momentum redistribution generates counterintuitive torque; vortex beams, where torque arises from the orbital angular momentum of helical phase structures; or spin-gradient light fields, where spatially varying spin angular momentum distributions lead to complex optical torque behaviour. In contrast, the mechanism demonstrated herein is governed by the geometric asymmetry of individual nonspherical particles under paraxial-trapping conditions. Particle morphology, encompassing shape and size, directly modulates scattering and absorption properties. These effects are further amplified by the off-axis trapping configuration, establishing an insightful framework for understanding and controlling optical torques and forces in paraxial optical systems.

## Results

In this work, we delve more deeply into this paraxial-trapping regime and uncover unexpected effects in the morphology- and rotation-induced optical torques (MRIOTs) and forces on shaped polystyrene particles. We find that a shaped particle, when slightly deflected from the beam centre in paraxial trapping, can exhibit counterintuitive effects on OTs that depend on its rotation angles and size in the light field. For the morphology in Fig. 1c, a negative OT emerges, whose value varies when the rotation changes. At a specific rotation angle, positive and negative OTs on a shaped particle can compete with each other and stabilise the rotation (Fig. 1d). Eventually, the particle can freeze in rotation and drift laterally by the optical lateral force^45–49^. Notably, the rotation angle can also induce reversible optical forces.

To investigate the MRIOTs and optical forces, we expose particles of various shapes and sizes to a focused, line-shaped spin light beam. This approach simplifies the 3D problem into a 2D scenario by converting the orbital rotation into lateral motion, thereby greatly facilitating the observation of intriguing OTs and optical forces within the same plane. Instead of continuous rotation of shaped particles by the OT in a spin field as showcased in previous studies, particles with various shapes, e.g., cylinder, behave differently depending on their rotation angles, as shown in Fig. 1e. The particle can even rotationally freeze under the balance of positive and negative OTs.

This unconventional MRIOT and optical force ubiquitously exhibit in various shapes, as shown in Fig. 1f, g. The evolution of particle shape can be characterised by the vertex coefficient *α* (Fig. 1f and Supplementary Note 1). Beyond its role as a geometric descriptor, variations in *α* influence particle polarizability, scattering behaviour, and optical field symmetry, which collectively mediate changes observed in optical torque and force, which eventually give rise to distinct positive and negative OTs. Those OTs also vary significantly with the rotation angle of the particle, as shown in Fig. 1f. Our work completes the missing building block in optical manipulation, highlighting the profound influence of particle morphology on optical forces and torques. These findings enrich the fundamental understanding of light–matter interactions and offer promising applications in optical sorting and advanced optical manipulation.

**Fig. 2:**
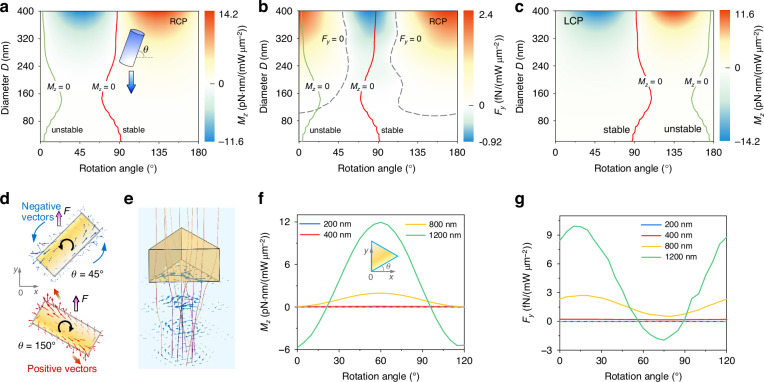
Simulation of morphology- and rotation-induced optical torques and forces. Dependence of the **a** OT and **b** optical force on the radius and rotation angle (*θ*) of the micro-cylinder when the incident light is RCP. The length-diameter ratio of the micro-cylinder is 2.5. **a** Two contours occur in the map, showing stable and unstable equilibrium positions (angles) for positive and negative OTs. **b** Stable angles correspond to negative optical forces, making the micro-cylinder move towards the −*y* direction. **c** OT versus the radius and rotation angle of the micro-cylinder when the incident light is left-handed circular polarised (LCP, polarisation angle *φ* = 135°). **d** Plot of force vectors surrounding the micro-cylinder (*D* = 200 nm) under the RCP light when *θ* = 45° and 150°, showing counter-clockwise (negative OT) and clockwise (positive OT) rotations of the micro-cylinder, respectively. Negative and positive vectors denote vectors most likely generating negative and positive OTs, respectively. **e** The energy flow (red lines) from the top passing through the triangular prism induces vortex-like Poynting vector arrows (blue arrows, only showing components in *x* and *y* directions) on different planes. The side and height of the triangular prism are 1200 and 600 nm, respectively. **f** OT and **g** optical force on various triangular prisms as a function of the rotation angle under the RCP light. It is seen that the rotation of the particle can reverse both the OT and optical force when the particle is large, e.g., side equals 1200 nm, showing the important role of the morphology. In (**e**, **f**), the centre of the triangular prism is located at *x* = 200 nm. In (**a**–**g**), *w*_*x*_ = 1000 nm and *w*_*y*_ = 500 μm. OTs are calculated by normalising the intensity at (*x* = 0, *y* = 0) to 1 W μm^−2^

To calculate the OT and optical force numerically, a circularly polarised light beam (wavelength *λ* = 532 nm) is focused into the line shape and projected onto the *x*-*y* plane (Fig. 1e). The focal widths in *x* and *y* directions are 1 and 500 µm, respectively. For a cylinder (length-diameter ratio is 2.5) placed at *x* = 200 nm (*x* = 0 is the beam axis), the OT is determined by the radius and rotation angle *θ* (length direction with respect to the *x*-axis) of the particle, as shown in Fig. 2a. The offset at *x* = 200 nm is critical in breaking the left-right symmetry of the optical field, enabling non-uniform force distributions and resulting in the generation of negative optical torques. The simulation is conducted in the commercial software COMSOL based on the Minkowski stress tensor. Specifically, the optical force can be given as^47,50^

1 $${\bf{F}}={\oint }_{s}\overleftrightarrow{{\bf{T}}}\cdot \hat{{\bf{n}}}dS$$

where $\hat{{\bf{n}}}$ is the unit vector outward normal to the integral surface, $\overleftrightarrow{{\bf{T}}}$ is the time-averaged Maxwell stress tensor in Minkowski form, which can be expressed as

2 $${T}_{ij}=\frac{1}{2}\left[{D}_{i}{E}_{j}^{\ast }+{B}_{i}{H}_{j}^{\ast }-\frac{1}{2}({\bf{D}}\cdot {{\bf{E}}}^{\ast }+{\bf{B}}\cdot {{\bf{H}}}^{\ast }){\delta }_{ij}\right]$$

where ${\delta }_{ij}$ is the Kronecker delta. Meanwhile, the optical torque **M** can be expressed as

3 $${\bf{M}}={\oint }_{s}({\bf{r}}\times \overleftrightarrow{{\bf{T}}})\cdot \hat{{\bf{n}}}dS$$

For the OT under RCP light, there are regions of positive and negative values with different parameters, as shown in Fig. 2a. Two contours denote *M*_*z*_ = 0, in which case, the cylinder moves laterally without rotating. It is worth mentioning that the system in a liquid environment stays in the overdamped regime, in which particles suspended in a viscous fluid rotate with a determined angular velocity driven by the optical torque. Within this regime, inertial contributions are negligible compared with the viscous drag from the ambient fluid, leading to a direct proportionality between the optical torque and the particle’s angular velocity. Accordingly, the particle rotation may stop at zero optical torque. However, the left contour represents unstable angles since the particle can rotate further away from the equilibrium point when *θ* slightly deviates. In contrast, the right contour represents stable angles as OTs can rotate the particle back to those points. Stable angles are located in the region with negative optical forces (*F*_*y*_ < 0), indicating that the micro-cylinder can move steadily towards the −*y* direction, as shown in Fig. 2b. Similar phenomena are observed for smaller or larger offset values (*x* = 100 and 300 nm or different positions, as shown in Fig. S7), where distributions of the optical torque and force exhibit the same behaviour but with different magnitudes due to the different intensity gradient associated with the different offset. Due to the system symmetry, optical forces and OTs under LCP light (polarisation angle *φ* = 135°, see Fig. 2c) are linked to those under RCP light (*φ* = 45°) with the following equations:

4 $$\begin{array}{c}{F}_{\varphi {=135}^{\circ }}(\theta )=-{F}_{\varphi {=45}^{\circ }}({180}^{\circ }-\theta )\\ {M}_{\varphi {=135}^{\circ }}(\theta )=-{M}_{\varphi {=45}^{\circ }}({180}^{\circ }-\theta )\end{array}$$

Positive, zero and negative OTs on the 100-nm-diameter micro-cylinder can be visualised by plotting force vectors on the surface of the particle, as shown in Fig. 2d. Force vectors calculated using the Minkowski stress tensor tend to rotate the cylinder clockwise and counter-clockwise, indicating positive and negative OTs when *θ* = 150° and 45°, respectively. It can be found that the negative OT mainly arise from the force vectors enclosing the micro-cylinder, which are also defined as the negative vectors. In contrast, positive vectors that point outward from the micro-cylinder contribute to the positive OT. Meanwhile, OTs equalling zero come from the balance of positive and negative vectors, as shown in Figs. S11 and S12. The dominance of negative vectors at *θ* = 45° and positive vectors at *θ* = 150° is attributed to the interplay between the particle’s morphology and its orientation relative to the light field. The particle’s geometric asymmetry modulates the scattering field distribution, redistributing optical momentum and resulting in positive and negative vectors with opposite signs at different rotation angles. Upon scattering by the triangular prism, part of the SAM of the incident light is converted into OAM of the scattered field, exerting a significant torque on the particle, as shown in Fig. 2e.

Figure 2f, g plot OTs and optical forces on equilateral triangles (2D view of the triangular prism) when the rotation angle and size are changed. It is shown that small triangles cannot generate negative OTs. In contrast, the 1200-nm-side triangle exhibits both reversible OTs (Fig. 2e) and optical forces (Fig. 2f), depending on different *θ*. This is because a small triangle can be treated as a dipole particle, whose OT follows the direction of the SAM. Large particles interact with the light field profoundly, which also mitigates the influence of the Brownian motion in the experiment, facilitating the observation of unexpected morphology-induced phenomena.

**Fig. 3:**
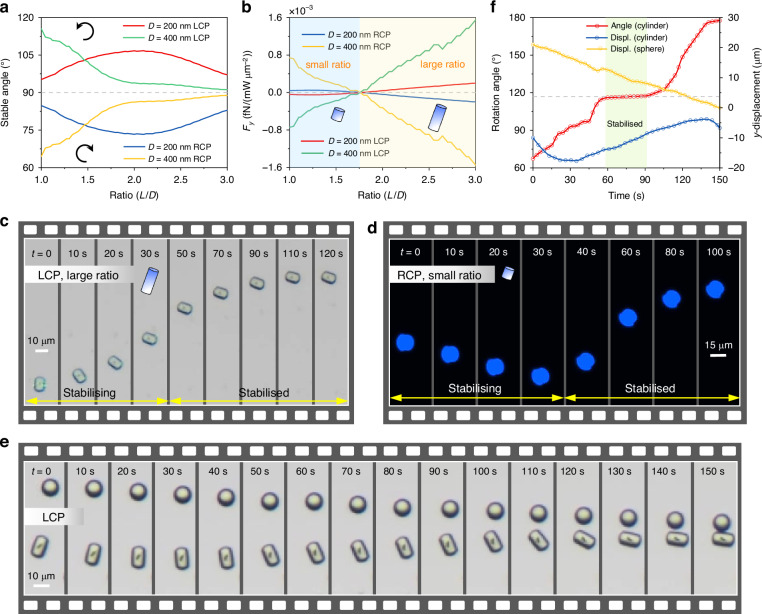
Observation of reversible morphology- and rotation-induced optical torques and forces. **a** Simulation of stable angles and **b** corresponding optical forces versus the length-diameter ratio of the micro-cylinder. The stable angle *θ*_stable_ > 90° or < 90° when the beam is LCP or RCP, respectively, which is also observed in (**c**–**e**). **b** The optical force can also be reversed when changing the ratio. Stabilising processes for **c** long and **d** short micro-cylinders when light beams are LCP (Supplementary Movie S1) and RCP (Supplementary Movie S2), respectively. *θ*_stable_ ~160° for **c** the LCP and *θ*_stable_ ~50° for **d** the RCP, respectively. The large ratio under the LCP light in (**c**) and the small ratio under the RCP light in (**d**) result in the movement both in the +*y* direction, as indicated in (**b**). **e** The sphere and micro-cylinder move towards opposite directions by distinct optical forces (Supplementary Movie S3). *θ*_stable_ ~125° for the micro-cylinder. **f** Retrieved trajectories and rotation angles for the sphere and micro-cylinder, indicating the stable optical force for the sphere and the MRIOT and optical force for the micro-cylinder. Laser powers in (**c**–**f**) are 500 mW

The plots of stable angles for various cylinders with different diameters and length-diameter ratios are shown in Fig. 3a. Corresponding comprehensive OT contour plots and optical force curves are shown in Figs. 3b and S8a−d, respectively. It can be seen that stable angles for RCP and LCP are mainly located in regimes with *θ* < 90° and *θ* > 90°, respectively. Two curves for RCP and LCP exhibit symmetry at *θ* = 90°. Though calculated using sub-microparticles due to the calculation constraint of the workstation, the large stable angle *θ* > 90° for the LCP and small stable angle *θ* < 90° for the RCP are also observed using micro-cylinders experimentally, as shown in Figs. 3c−e and S13. The micro-cylinder with a diameter of 6.2 µm and a length of 11 µm started to rotate since it was initially under an unstable rotation angle, as shown in Fig. 3c and Supplementary Movie S1. The particle under the LCP light gradually rotated to its stable angle (~155°) and steadily moved upwards (+*y* direction) until the upper edge of the light beam. In contrast, the fluorescence micro-cylinder under the RCP light has a stable angle of ~50°, as shown in Fig. 3d and Supplementary Movie S2. The small angle, i.e., *θ* < 90°, can also be indicated in Fig. 3a. Intuitively, the micro-cylinder in Fig. 3d moves in opposite *y* directions with that in Fig. 3c due to the opposite helicity of light. However, they both move in the +*y* direction because of small and large length-diameter ratios, as indicated in Fig. 3b. More experimental results about the small and large length-diameter ratios can be found in Fig. S17, which also coincide well with the prediction in Fig. 3a, b.

With the influence of rotation angle on the optical force and OT, the micro-cylinder may rotate and locomote in the opposite *y* direction with a spherical particle, as shown in Fig. 3e and Supplementary Movie S3. The 10-µm polystyrene particle moves steadily in the −*y* direction with a constant velocity, indicating a stable optical force, which can be obtained using the equation *F*_opt_ = *F*_drag_ = 6*πηav*, where *η* is the viscosity of the liquid and *v* is the velocity of the particle. The rotation angle and displacement of the micro-cylinder as a function of time are plotted in Fig. 3f. The micro-cylinder began to be stabilised after 60 s. However, its stabilisation was disturbed by the microsphere at ~90 s, resulting in the binding of two particles^51^.

**Fig. 4:**
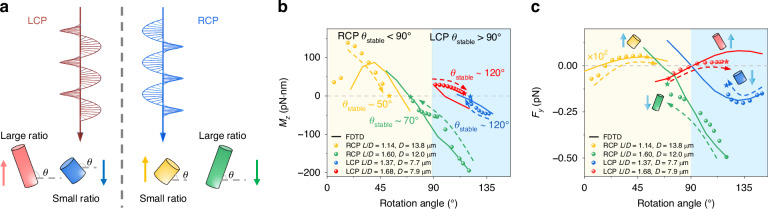
Experimental and simulated comparisons of the forces and torques experienced by particles in the optical field. **a** A depiction of the dynamical behaviour of particles in an optical field. Experimental (scatter) and FDTD simulation (solid line) curves of **b** OTs and **c** optical forces versus the rotation angle

To further clarify how particle ratio and light polarisation affect OTs and optical forces, we compare micro-cylinders with different length-to-diameter ratios under RCP and LCP light illumination (Fig. 4). Both small- and large-ratio cylinders show the same polarisation-dependent rotational behaviours (Fig. 4a): RCP light stabilises the cylinder at *θ*_stable_ < 90° but LCP light stabilises the cylinder at *θ*_stable_ > 90°. Meanwhile, when rotationally stabilised, cylinders with a small ratio move upwards under RCP light and downwards under LCP light. By contrast, cylinders with a large ratio move upwards under LCP light and downwards under RCP light. These trends are consistently demonstrated by rigorous FDTD simulations and experimental measurements (Fig. 4b, c, more details can be found in Materials and methods).

**Fig. 5:**
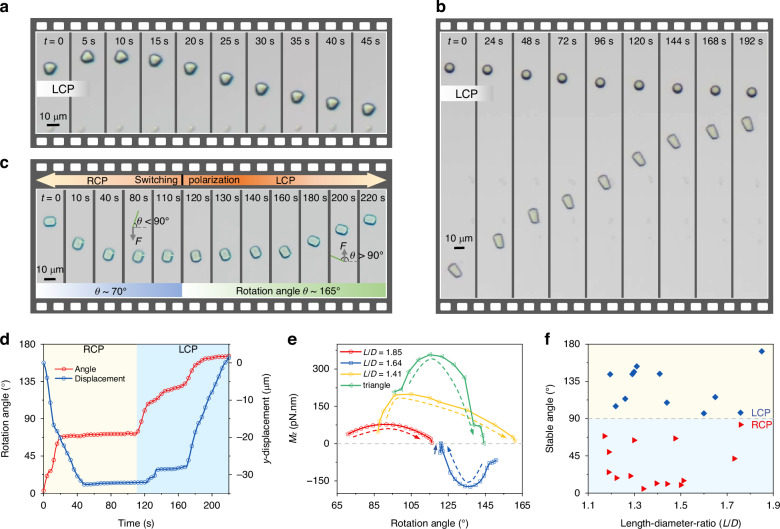
Shape-, rotation- and polarisation-dependent optical torques and forces. **a** Stabilising process of the triangular prism, which moves downwards (−*y* direction) when the rotation becomes stabilised (Supplementary Movie S4). This movement is similar to the sphere in (**b**). **b** The special-shaped particle has a similar behaviour to that of the micro-cylinder (Supplementary Movie S5). **c** Control of OTs and optical forces with the light polarisation. The rotation angle changes and the moving direction reverses when the light polarisation is changed from the RCP to LCP (Supplementary Movie S6). This is also observed from the retrieved plot of rotation angle and displacement in (**d**). **e** Measured OTs versus the rotation angle. The OT can be positive and negative depending on the initial rotation angle. Particles gradually rotate to their stable angles, which are related to their shapes and length-diameter ratios. **f** Measured stable angles for micro-cylinders with various length-diameter ratios under the LCP and RCP light. Stable angles for the LCP and RCP exhibit in regimes of *θ* > 90° and *θ* < 90°, respectively. Laser powers in (**a**–**f**) are 500 mW

We then investigate the influence of particle shape, size (length-diameter ratio) and light polarisation on the OT and optical force. Akin to the micro-cylinder, the triangle also has a stable angle in the light field, as shown in Fig. 5a and Supplementary Movie S4. The triangle can either move upwards (+*y* direction) or downwards (−*y* direction), depending on different rotation angles. The stable angle gives rise to a downward movement and a negative optical lateral force. The particle with a trapezoid shape in Fig. 5b (see Supplementary Movie S5) has a similar behaviour to the micro-cylinder in Fig. 3c. Distinctively, the trapezoid-shaped particle has a smaller stable angle of ~120°.

When the light polarisation is changed from the RCP to the LCP, it can be expected from Eq. (4) that the OT and optical force can change significantly. Specifically, the rotation angle starts to change when the light handedness is reversed, and the moving direction becomes opposite, as shown in Fig. 5c, d, as well as Supplementary Movie S6. The stable angles for RCP and LCP are <90° and >90°, respectively. These, along with the opposing directions of motion, emphasise the pronounced effect of light polarisation on optical torque and force. Experimentally, each shaped particle exhibits a specific stable angle, as shown in Fig. 5e. Particles gradually rotate to their stable angles under the influence of positive and negative optical torques, depending on their initial states. Sometimes, the particle rotates away from the stable angle because of the inertia or disturbance, while it can rotate back to that point swiftly, as shown in the blue curve in Fig. 5e. Measured stable angles for micro-cylinders with various length-diameter ratios under the LCP and RCP are plotted in Fig. 5f. Aligned with simulation results in Fig. 3a, stable angles for the LCP and RCP are maintained in regimes of *θ* > 90° and *θ* < 90°, respectively. Although Figs. 3a and 5f focus on sub-microparticles and microparticles, respectively, both results align with the general trend (*θ* > 90° for the LCP and *θ* < 90° for the RCP) observed in the two regimes. Regardless of variations in particle size, rotation angle, position, refractive index, or the polarisation state of the light (LCP or RCP), rotational freezing can be observed in the particles, which ultimately undergo lateral motion. Therefore, this can be considered a generic phenomenon instead of a coincidental one. Besides, more simulation results can be found in the Supplementary Materials.

## Discussion

Particles with distinctive morphologies provide versatile opportunities for diverse physical and biological applications. In contrast to spheres, shaped particles exhibit different dynamics in the light field. Exploiting their OTs and optical forces requires a deep understanding of the intriguing light–matter interaction. This work delves into an overlooked and mysterious field in which particle morphology and rotation could induce counterintuitive OTs and optical forces. This happens ubiquitously in a paraxial system with the inhomogeneous SAM. It is also worth noting that the paraxial system is exhibited widely in optical manipulation, where the optical radiation pressure pushes away the microparticle from the focal point, balancing with the optical gradient force. This effect becomes more profound when manipulating the microparticle on a slightly defocused plane, which results in paraxial trapping.

The OT and optical force are strongly dependent on the morphology and rotation angle of the particle, contrasting with previous assumptions that overlook the subtle asymmetry of the light field and its impact on the shaped particle. The influence of particle morphology on optical torque and force may be linked to the excitation of higher-order multipole modes, which modulate scattering asymmetry. These modes could potentially be sensitive to a particle’s geometry, aspect ratio, and vertex distribution, with changes in these morphological traits altering the angular distribution of the scattered field. While multipole interactions are related to the scattering response, variations in optical torque and force are further governed by how particle morphology interacts with the optical field. Structural asymmetry dynamically modifies the particle’s coupling with the light field, redistributing optical momentum and determining the torque’s direction and magnitude. For instance, with different rotation angles, the optical force and OT can reverse their signs. Instead of continuously rotating particles in a spin light field, shaped particles can freeze under the balance of negative and positive OTs. Stable angles under rotational freezing are determined by the shape and size of particles. The intriguing reversible forces and torques can be utilised for shape-based sorting, providing a precious tool for optical manipulation.

Beyond rigid particles applied in our experiments (e.g. polystyrene), the optical forces and torques on non-rigid particles (e.g., biological particles) are also strongly influenced by their shape and morphology. However, unlike rigid particles, non-rigid particles could possibly deform under optical forces, dynamically altering their effective geometry and rotational symmetry. This deformation enhances asymmetry in light–matter interactions, leading to shape-dependent optical effects that may amplify or diminish forces and torques. In most research, non-rigid behaviour of bioparticles is either not taken into consideration or regarded as insignificant^52,53^.

Our work enriches the conventional understanding of light–matter interaction by exploiting the intriguing MRIOT and optical force. Exploiting important roles of particle morphology and rotation may pave the way for advanced optical manipulation, optical sorting and binding, endowing significant applications in biophysics, optofluidics, quantum science and metaoptics.

## Materials and methods

### Numerical simulations of the optical force and torque

Simulations of optical force and OT are conducted in the commercial software COMSOL using the Minkowski stress tensor, as expressed in Eqs. (1–3). The light field used in the simulation is a focused line-shaped beam which can be expressed as

5 $${\bf{E}}=A\left[\cos \,\varphi \bar{{\bf{x}}}+i\,\sin \,\varphi \bar{{\bf{y}}}+\frac{2i}{k}\left(\frac{x}{{w}_{x}^{2}}\,\cos \,\varphi +\frac{iy}{{w}_{y}^{2}}\,\sin \,\varphi \right)\bar{{\bf{z}}}\right]\exp\left[-\left(\frac{{x}^{2}}{{w}_{x}^{2}}+\frac{{y}^{2}}{{w}_{y}^{2}}\right)\right]\exp (-ikz)$$

where *A* is the wave amplitude, $\bar{{\bf{x}}}$, $\bar{{\bf{y}}}$ and $\bar{{\bf{z}}}$ are unit vectors of corresponding axes; *k* is the wavenumber in the medium; *φ* is the polarisation angle, which is the angle between the electric field and the fast axis of the quarter-wave plate (*x*-axis); *w*_*x*_ and *w*_*y*_ are the half focal widths in *x* and *y* directions, respectively. In this work, *w*_*x*_ and *w*_*y*_ are set to 1 and 500 µm, respectively. The long focal length in the *y*-direction ensures that the particle experiences negligible optical gradient force, and the focal length in the *x*-direction traps the particle to mitigate the drift of the particle in this direction.

### General formula of optical torques on various particles

Optical torques on whether spherical or nonspherical particles, symmetric or asymmetric particles, their OTs can be expressed by a more general formula:

6 $${M}_{z}=-\frac{\varepsilon }{2{k}^{3}}\mathop{\sum }\limits_{l=1}^{N}\mathop{\sum }\limits_{m=-l}^{l}m[{\mathrm{Re}}({q}_{ml}{b}_{ml}^{\ast }+{p}_{ml}{a}_{ml}^{\ast })+{|{q}_{ml}|}^{2}+{|{p}_{ml}|}^{2}]$$

where *ε* is the permittivity of the medium, $k=n\omega /c$ is the wavenumber in the medium, *l* is the order of the multipole expansion, *m* is the magnetic quantum number for each order of the multipole moment, ${p}_{{ml}}$ and ${q}_{{ml}}$ correspond to the expansion coefficients of the vector spherical harmonic functions (VSHFs) for the scattered field, ${a}_{{ml}}$ and ${b}_{{ml}}$ correspond to the expansion coefficients of the VSHFs for the incident field, ${a}_{{ml}}$, ${b}_{{ml}}$, ${p}_{{ml}}$, and ${q}_{{ml}}$ are related through the T-matrix (see Supplementary Note 2 for details).

As the rotation angle of the particle changes and its symmetry begins to break, i.e., when the rotation angle of the particle starts to vary, elements of the T-matrix are correspondingly affected. The contribution of the off-diagonal terms in the T-matrix becomes significant, leading to the emergence of positive or negative torques, or torques with a zero value.

### Experimental setup and configurations

The experimental setup is the same as the one used in our previous study that investigates optical lateral forces on microspheres^47^. The line-shaped light beam is projected into a microscopic system using two cylindrical lenses with focal lengths of 400 and 30 mm. Microparticles (Jike Biotechnology Col., Ltd.) are diluted into deionised water, which is then dropped into a chamber on the cover slide. In the experiments, the focused beam spot has a width of ~20 μm along the *x*-direction and a length of ~300 μm along the *y*-direction. The chamber is fabricated by sticking the imaging gasket with a cylindrical hole (9 mm in diameter and 0.12 mm in depth) on the cover slide. After that, another cover slide is used to seal the chamber. Microparticles with different shapes, such as triangles, cylinders and trapezoids are picked from a large number of imperfect sphere particles due to the synthesised impurity. The quantity of rod-shaped and specially shaped particles is much smaller compared to microspheres. Experiments are conducted when particles are stabilised from the background environment. Polarisations are controlled using the quarter-wave plate. Images of particles are observed using a ×10 objective lens and recorded with a camera (Nikon, Digital Sight 10). The wavelength of the laser is 532 nm.

### Experimental calculations of optical torques and forces

During each small period of time in the experiment, the particle rotates uniformly around its centre. For spherical particles^54^, their OTs can be calculated using *τ* = −8*πηa*^3^*Ω*, where *η* is the viscosity of the liquid (10^−3^ Pa·s for water), *a* and *Ω* represent the radius and rotation velocity of the particle, respectively. For cylindrical particles^55^, their OTs can be calculated using $\tau \,=\,-\varOmega \pi \eta {L}^{3}/\{3[\mathrm{ln}(L/D)+1/\,\mathrm{ln}(2L/D)]\}$, where *L* and *D* are the height and the diameter of the cylinder, respectively. Optical forces on the cylinder particles^56^ in the y direction can be obtained using ${F}_{{\rm{op}}{{\rm{t}}}_{y}}={F}_{{\rm{dra}}{{\rm{g}}}_{y}}=4\pi \eta {vL}{\sin }^{2}\theta /[\mathrm{ln}(2L/D)+\mathrm{ln}2-0.5]+2\pi \eta {vL}{\cos }^{2}\theta /[\mathrm{ln}(2L/D)+\mathrm{ln}2-1.5]$, where *L* and *D* are the height and the diameter of the cylinder, respectively, *θ* is the rotation angle of the particle. Velocities are calculated from retrieved trajectories of particle centres.

### FDTD simulations of optical forces and torques

Simulations of optical forces and OTs on nano-sized particles are conducted in COMSOL, while simulations of them on micron-scale cylindrical particles are conducted in the commercial software Ansys Lumerical FDTD, both using the Minkowski stress tensor [Eqs. (1–3) in the main text]. We employ the FDTD method in Lumerical to complement the FEM method in COMSOL. FDTD enables large-area simulations (>10 wavelengths), which support direct comparisons between theoretical and experimental results (Fig. 4b, c). The light field used in the simulation is a focused line-shaped beam as expressed in Eq. (S1). To closely replicate the line-shaped light field produced in the experiment, *w*_*x*_ and *w*_*y*_ are set to ~15 and ~500 μm, respectively. The line-shaped Gaussian light field was imported into the FDTD simulation domain via a script. The dimensions of the simulation domain were set to 50, 120, and 30 μm along the *x*, *y*, and *z* axes, respectively. Four particles matching experimental sizes are simulated using circularly polarised light identical to experimental conditions. Length *L*, diameter *D* and light polarisation for the four particles are 13.3 μm, 7.9 μm, LCP; 10.5 μm, 7.7 μm, LCP; 19.3 μm, 12.0 μm, RCP; 15.8 μm, 13.8 μm, RCP, respectively. The boundary conditions are implemented using a perfectly matched layer. The maximum mesh within the particle was set to 0.08 μm in *x*, *y*, and *z* directions. Both small and large particles come to the same conclusion: (1) Particle morphology and rotation significantly impact the OT and optical force; Stable angle *θ*_stable_ < 90° and *θ*_stable_ > 90° for RCP and LCP light, respectively (Fig. 4b); (2) When rotationally stabilised, cylinders with a small ratio under RCP light and those with a large ratio under LCP light move upwards, whereas cylinders with a small ratio under LCP light and those with a large ratio under RCP light move downwards (Fig. 4c).

## Supplementary information

Supplementary Material for Impacts of particle morphology and rotation on optical manipulation

Movie S1 Stabilising process of a long micro-cylinder when the light is LCP

Movie S2 Stabilising process of a short micro-cylinder when the light is RCP

Movie S3 Bilateral movement and rotation of a sphere and a micro-cylinder

Movie S4 Stabilising process of a triangular prism when the light is LCP

Movie S5 Bilateral movement and rotation of a sphere and a special-shaped particle

Movie S6 Polarisation-dependent bilateral movement and rotation of a micro-cylinder

## Data Availability

The data that support the findings of this study are available from the corresponding authors upon reasonable request.
