# Supplementary material for: Impacts of particle morphology and rotation on optical manipulation
Source: Light Sci Appl. 2026 Jul 13;15:313. doi: 10.1038/s41377-026-02403-5 (PMC13357792; doi:10.1038/s41377-026-02403-5)
Supplement: Supplementary file 1 — Supplementary Material for Impacts of particle morphology and rotation on optical manipulation [file 41377_2026_2403_MOESM1_ESM.pdf]

Supplementary Material for
Impacts of particle morphology and rotation on optical manipulation

Weicheng Yi[1,2,3,4,5#], Yuzhi Shi[1,2,3,4#*], Hongfei Jiao[1,2,3,4*], Chengxing Lai[1,2,3,4],
Haiyang Huang[1,2,3,4], Xinhua Dai[5], Xiaoyun Gong[5], Hui Zhang[1,2,3,4],
Qinghua Song[6], Zhanshan Wang[1,2,3,4], Zeyong Wei[1,2,3,4],
C. T. Chan[7], Cheng-Wei Qiu[8*] and Xinbin Cheng[1,2,3,4*]

[1] Institute of Precision Optical Engineering, School of Physics Science and Engineering, Tongji University, Shanghai 200092, China

[2] MOE Key Laboratory of Advanced Micro-Structured Materials, Shanghai 200092, China.

[3] Shanghai Institute of Intelligent Science and Technology, Tongji University, Shanghai 200092, China

[4] Shanghai Frontiers Science Center of Digital Optics, Shanghai 200092, China

[5] Technology Innovation Center of Mass Spectrometry for State Market Regulation, Center for Advanced Measurement Science, National Institute of Metrology, Beijing 100029, China;

[6] Tsinghua Shenzhen International Graduate School, Tsinghua University, Shenzhen 518055, China

[7] Department of Physics, The Hong Kong University of Science and Technology, Clear Water Bay, Kowloon, Hong Kong, China

[8] Department of Electrical and Computer Engineering, National University of Singapore, Singapore 117583, Singapore

#Those authors contribute equally to this paper.

*Corresponding authors: yzshi@tongji.edu.cn (Y.S.); jiaohf@tongji.edu.cn (H.J.); chengwei.qiu@nus.edu.sg (C.-W.Q.); chengxb@tongji.edu.cn (X.C.)

Supplementary Note 1: construction process of geometric and physical models

For both spherical and ellipsoidal models, the geometric centers of the sphere and ellipsoid are placed at the origin of the coordinate system. For the sphere, its radius is set to 218.5 nm to make the area 0.15 μm^2, and the optical torque (OT) M_z (rotation axis is the z-axis) exerted on the sphere in the optical field is calculated using the Minkowski stress tensor method. Since M_z on an isotropic, homogeneous dielectric particle remains invariant regardless of its rotation, no additional parameters are required to compute M_z for different rotational angles of the sphere. For the ellipsoid, the lengths of the semi-major axis, semi-minor axis, and semi-intermediate axis are set to 238.73 nm, 200 nm, and 200 nm, respectively. The semi-major axis, semi-minor axis, and semi-intermediate axis are aligned with the x-, y- and z-axes, respectively. The projection of the ellipsoid onto the x-y plane forms an ellipse. The parameter θ is defined as the counter-clockwise rotation angle of the ellipse in the x-y plane about the origin. The OT exerted on the ellipsoid is then calculated as θ varies from 0° to 180°. Schematic diagrams of the sphere and ellipsoid in the x-y plane are shown in Figs. S1a and S1g, respectively.

For the cylindrical model, the geometric center is also positioned at the origin of the coordinate system, with its base oriented perpendicular to the z-axis. Consequently, the projection of the cylinder onto the x-y plane forms a rectangle. Two cylindrical models with different parameters are considered: (1) a cylinder with a base radius of 193.65 nm and a height of 387.30 nm, resulting in a square projection in the x-y plane; (2) a cylinder with a base radius of 150 nm and a height of 500 nm, resulting in a more general rectangular projection in the x-y plane. In the initial configuration, the diagonal of the square and rectangle is aligned with the x-axis, and the edges of the projection of the cylinder's base in the x-y plane are situated in the second and fourth quadrants. Similarly, we define the parameter θ to represent the counter-clockwise rotation angle of the rectangle and square about the origin in the x-y plane. The OT is calculated as θ varies from 0° to 180°. Schematic diagrams of the square and rectangular projections of two cylindrical models in the x-y plane are shown in Figs. S1b and S1h, respectively.

For the prismatic polygonal model, we first construct a two-dimensional polygon parallel to the x-y plane and at a distance of $h/2$ from it. This polygon is then extruded along the direction toward the origin by a length h, ensuring that the resulting prism is symmetric about the y-z plane. The polygon is defined as follows: in a two-dimensional coordinate system, the circumcenter of

the polygon (i.e., the center of its circumscribed circle) is set at the origin, with the circumradius denoted as R. For an n-sided polygon, n angular values $\theta_1, \theta_2, \ldots, \theta_n$ are selected. The coordinates of the i-th vertex are determined by $(R\cos\theta_i, R\sin\theta_i)$. By setting $\theta_1 = 0$, the first vertex lies on the positive x-axis, the specific process is illustrated in Fig. S2. The extrusion length h is set as half of the circumradius R. The parameters for different polygons are provided in Table S1. Similarly, we define the parameter θ_R to represent the counter-clockwise rotation angle of the projection of the prismatic polygon in the x-y plane about the origin. The schematic diagrams of projections of different prismatic polygons in the x-y plane are shown in Figs. S1c–S1f and S1i–S1l.

The configuration of all these parameters ensures that the projection areas of these three-dimensional shapes in the x-y plane are all 0.15 μm^2.

The vertex coefficient α is defined to distinguish different shapes of three-dimensional geometries:

$$\alpha = \frac{V + F}{10} \tag{S1}$$

where V represents the number of vertices in the top view of the three-dimensional geometry, and F represents the number of vertices in the side view of the three-dimensional geometry. The denominator 10 is chosen for normalization purposes, facilitating a systematic representation of the shape evolution from a sphere ($\alpha = 0$) to a hexagonal prism ($\alpha = 1$). The α values of different three-dimensional models are shown in Table S2.

As the sphere and ellipsoid start to evolve, i.e., as the vertex coefficient α increases from 0, the simulated OT curves for different shapes are shown in Figs. S3 and S4. With the increase of α, the geometric asymmetry of the particle becomes more pronounced, leading to modifications in the optical field symmetry and scattering directionality, which together govern the generation of positive and negative OTs. Normally, compared to regular prisms, general prisms exhibit larger torque magnitudes, accompanied by more pronounced oscillations in the magnitude variation. This difference is attributed to the superior symmetry of regular prisms. Furthermore, for the same prism (cylindrical or prismatic), OTs may exhibit a directional reversal as the rotation angle changes.

Although we minimized the torque variations caused by size differences by maintaining the

same area, it could not be completely eliminated. Therefore, to demonstrate that the torque variations in Figs. S3 and S4 indeed result from shape differences, we performed the following simulations.

We also investigated prisms with the same circumscribed radius but different vertex coefficients α to demonstrate that the variations in optical force and torque are not solely attributed to changes in particle size. In one set of simulations, we fixed the circumscribed radius of particles (300 nm, 400 nm, and 500 nm) and analyzed the evolution from cylindrical to hexagonal shapes (α = 0.4−1.0). The results revealed that even with the same circumscribed radius, shape evolution significantly affects the polarizability and, consequently, the optical field symmetry and scattering properties (e.g., scattering cross-section), leading to notable variations in optical torques and scattering cross-section, as shown in Fig. S6a.

While α provides a systematic approach for characterizing shape evolution, it does not fully account for the impact of vertex distribution. For example, particles with identical α values, such as equilateral and scalene triangles, can exhibit distinct scattering and optical torque characteristics owing to their vertex asymmetry. As a result, we also investigated the variations in optical torque for particles with the same α, the same circumscribed radius, but different vertex distributions. Triangular prisms with circumscribed radius of 300 nm, 400 nm, and 500 nm were selected, by fixing two vertex positions (θ_1 = 0°, θ_2 = 90°) and varying the position of the third vertex (θ_3 = 105°−225°), as shown in Fig. S6b. Our simulations demonstrate significant changes in scattering cross-sections and torque generation when altering vertex positions in triangular prisms with fixed circumscribed radius. For triangular prisms of the same shape and morphology but different circumscribed radius, the magnitude of the optical torque exhibits a positive correlation with the size of the prism. Conversely, for triangular prisms with the same circumscribed radius but different vertex distributions, those with higher symmetry tend to exhibit smaller torque magnitudes.

Supplementary Note 2: general formula of optical torques on various particles

The electric field of the line-shaped focused beam can be approximately written as:

$$\mathbf{E} = A[\cos\varphi\bar{\mathbf{x}} + i\sin\varphi\bar{\mathbf{y}} + \frac{2i}{k}(\frac{x}{w_x^2}\cos\varphi + \frac{iy}{w_y^2}\sin\varphi)\bar{\mathbf{z}}]\exp\left[-\left(\frac{x^2}{w_x^2} + \frac{y^2}{w_y^2}\right)\right]\exp(-ikz) \qquad \text{(S2)}$$

where A is the wave amplitude, $\bar{\mathrm{x}}$, $\bar{\mathbf{y}}$ and $\bar{\mathbf{z}}$ are unit vectors of corresponding axes; k is the wave number in the medium; φ is the polarisation angle, which is the angle between the electric field and the fast axis of the quarter-wave plate (x-axis); w_x and w_y are the half focal widths in x and y directions, respectively. $k = n\omega / c$ is the wavenumber in the medium, where $n = \sqrt{\varepsilon\mu}$ is the refractive index of the medium, ω is the frequency, and c is the speed of light.

Based on previous studies[1,2], it can be derived that under the dipole approximation, the torque acting on a spherical particle with uniform material composition throughout is:

$$\mathbf{M} = \frac{1}{2}\mathrm{Re}(\mathbf{d}_{es}^* \times \mathbf{E} + \mathbf{d}_{ms}^* \times \mathbf{H}) \qquad \text{(S3)}$$

where $\mathbf{d}_{es}$ and $\mathbf{d}_{ms}$ are the complex electric and magnetic dipole moments of the spherical particle, respectively, which can be described as

$$\mathbf{d}_{es} = \alpha_{es}\mathbf{E}, \qquad \mathbf{d}_{ms} = \alpha_{ms}\mathbf{H} \qquad \text{(S4)}$$

where α_{es} and α_{ms} are the complex electric and magnetic polarizabilities of the spherical particle, respectively. And α_{es} and α_{ms} can be written as[1,3]:

$$\alpha_{es} = \alpha_{es}^0(1 - i\frac{2}{3}k^3\alpha_{es}^0)^{-1}, \qquad \alpha_{ms} = \alpha_{ms}^0(1 - i\frac{2}{3}k^3\alpha_{ms}^0)^{-1} \qquad \text{(S5)}$$

where

$$\alpha_{es}^0 = \varepsilon a_s^3\frac{\varepsilon_p - \varepsilon}{\varepsilon_p + 2\varepsilon}, \qquad \alpha_{ms}^0 = \mu a_s^3\frac{\mu_p - \mu}{\mu_p + 2\mu} \qquad \text{(S6)}$$

where ε and μ are the permittivity and permeability of the medium, respectively, ε_p and μ_p are the permittivity and permeability of the particle, respectively, a_s is the radius of the spherical particle.

For non-spherical particles, applying the dipole approximation requires replacing the scalar polarizability with the corresponding polarizability tensor. The polarizability tensor must then be substituted into Eqs. (S3) and (S4) to obtain the optical torque. For a non-absorbing and non-magnetic dipole particle in a non-absorbing medium, only the electric dipole should be considered.

Taking an ellipsoidal particle as an example, consider an ellipsoid placed in its initial position as described in Note 1. Due to its symmetry, the off-diagonal elements are all zero, its polarizability tensor contains only diagonal components, and the corresponding components can be expressed by the following equations[4]:

$$\alpha_{eex}^{0} = \frac{V_e(\varepsilon_p - \varepsilon)}{\varepsilon + L_{ex}(\varepsilon_p - \varepsilon)}, \quad \alpha_{eey}^{0} = \frac{V_e(\varepsilon_p - \varepsilon)}{\varepsilon + L_{ey}(\varepsilon_p - \varepsilon)}, \quad \alpha_{eez}^{0} = \frac{V_e(\varepsilon_p - \varepsilon)}{\varepsilon + L_{ez}(\varepsilon_p - \varepsilon)} \tag{S7}$$

where V_e is the volume of the ellipsoidal particle, L_{ex}, L_{ey}, L_{ez} is the x, y, and z components of the depolarization factors, expressions for these parameters are as follows[5]:

$$V_e = \frac{4}{3}\pi a_e b_e c_e \tag{S8}$$

$$\begin{aligned} L_{ex} &= \frac{a_e b_e c_e}{2}\int_0^\infty \frac{1}{(s+a_e^2)\sqrt{(s+a_e^2)(s+b_e^2)(s+c_e^2)}}ds \\ L_{ey} &= \frac{a_e b_e c_e}{2}\int_0^\infty \frac{1}{(s+b_e^2)\sqrt{(s+a_e^2)(s+b_e^2)(s+c_e^2)}}ds \\ L_{ez} &= \frac{a_e b_e c_e}{2}\int_0^\infty \frac{1}{(s+c_e^2)\sqrt{(s+a_e^2)(s+b_e^2)(s+c_e^2)}}ds \end{aligned} \tag{S9}$$

where a_e, b_e, c_e are the semi-major axis, semi-minor axis, and semi-intermediate axis of the ellipsoidal particle.

Thus, we have derived the polarization tensor of the ellipsoid without radiative correction as

$$\vec{\vec{\alpha}}_{ee}^{0} = \begin{bmatrix} \alpha_{eex}^{0} & 0 & 0 \\ 0 & \alpha_{eey}^{0} & 0 \\ 0 & 0 & \alpha_{eez}^{0} \end{bmatrix} \tag{S10}$$

The polarization tensor of the ellipsoidal particle after radiative correction is[2]:

$$\vec{\vec{\alpha}}_{ee} = \vec{\vec{\alpha}}_{ee}^{0} \cdot [\vec{\vec{\mathbf{I}}} - \frac{2}{3} i k^3 \vec{\vec{\alpha}}_{ee}^{0}] \tag{S11}$$

where $\vec{\mathbf{I}}$ is the three-dimensional identity matrix.

Then the complex electric dipole moments of the ellipsoidal particle can be written as

$$\mathbf{d}_{ee} = \vec{\alpha}_{ee} \cdot \mathbf{E} \tag{S12}$$

When the ellipsoidal particle rotates counter-clockwise by an angle θ, the new polarization tensor without radiative correction can be obtained by multiplying the original polarization tensor with the rotation matrix as

$$\vec{\alpha}_{ee}^{0\,'} = R(\theta) \cdot \vec{\alpha}_{ee}^{0} \cdot R(\theta)^{T} \tag{S13}$$

where $R(\theta)$ is the rotation matrix and $R(\theta)^T$ is the transpose of the rotation matrix, they can be written as

$$R(\theta) = \begin{bmatrix} \cos\theta & -\sin\theta & 0 \\ \sin\theta & \cos\theta & 0 \\ 0 & 0 & 1 \end{bmatrix}, \qquad R(\theta)^{T} = \begin{bmatrix} \cos\theta & \sin\theta & 0 \\ -\sin\theta & \cos\theta & 0 \\ 0 & 0 & 1 \end{bmatrix} \tag{S14}$$

In this case, the polarization tensor of the ellipsoid is no longer applicable to the aforementioned radiative correction as the polarization tensor now contains off-diagonal terms. Higher-order radiative responses need to be considered, making it difficult to provide an analytical form for the polarization tensor of the ellipsoidal particle. Similarly, when using the polarization tensor instead of the scalar polarizability to handle some symmetric non-spherical particles, such as cylinders or regular prisms, the aforementioned issues will also arise. Therefore, if one still intends to use the dipole approximation to calculate the theoretical optical torque, numerical simulations must be employed to obtain numerical solutions.

Nevertheless, when encountering the vast diversity of particle morphologies found in nature, such a methodology would no longer be appropriate. To address the issue of anisotropy arising from the asymmetry in particle morphology and rotation, we need to consider multipole moments and adopt a more general form of optical torque[6,7], as provided in ref.[6]:

$$M_z = -\frac{\varepsilon}{2k^3} \sum_{l=1}^{N} \sum_{m=-l}^{l} m[\mathrm{Re}(q_{ml} b_{ml}^{*} + p_{ml} a_{ml}^{*}) + |q_{ml}|^2 + |p_{ml}|^2] \tag{S15}$$

where l is the order of the multipole expansion, m is the magnetic quantum number for each order of the multipole moment, p_{ml} and q_{ml} correspond to the expansion coefficients of the vector

spherical harmonic functions (VSHFs) for the scattered field, a_{ml} and b_{ml} correspond to the expansion coefficients of the VSHFs for the incident field, a_{ml}, b_{ml}, p_{ml}, and q_{ml} are related through the T-matrix[8]:

$$p_{ml} = \sum_{l'=1}^{N} \sum_{m'=-l'}^{l'} (T^{11}_{mlm'l'} a_{m'l'} + T^{12}_{mlm'l'} b_{m'l'})$$
$$q_{ml} = \sum_{l'=1}^{N} \sum_{m'=-l'}^{l'} (T^{21}_{mlm'l'} a_{m'l'} + T^{22}_{mlm'l'} b_{m'l'}) \quad \text{(S16)}$$

$$a_{ml} = 4\pi(-1)^m i^l G_{ml} \mathbf{E} \cdot \mathbf{C}_{-ml}(\vartheta, \phi)$$
$$b_{ml} = 4\pi(-1)^m i^{l-1} G_{ml} \mathbf{E} \cdot \mathbf{B}_{-ml}(\vartheta, \phi) \quad \text{(S17)}$$

where

$$G_{ml} = \sqrt{\frac{(2l+1)(l+m)!}{4\pi(l+1)(l-m)!}} \quad \text{(S18)}$$

VSHFs are given by

$$\mathbf{B}_{lm}(\vartheta, \phi) = [\mathbf{e}_\vartheta \frac{d}{d\vartheta} P_l^m(\cos\vartheta) + \mathbf{e}_\phi \frac{im}{\sin\vartheta} P_l^m(\cos\vartheta)] e^{im\phi}$$
$$\mathbf{C}_{lm}(\vartheta, \phi) = [\mathbf{e}_\vartheta \frac{im}{\sin\vartheta} P_l^m(\cos\vartheta) - \mathbf{e}_\phi \frac{d}{d\vartheta} P_l^m(\cos\vartheta)] e^{im\phi} \quad \text{(S19)}$$

For spherical particles, the T-matrix contains only diagonal elements (T^{11}, T^{22}), and the above incident and scattering coefficients reduce to the Mie coefficients[9]. However, for non-spherical particles, due to their vast diversity of particle morphologies, the off-diagonal elements (T^{12},T^{21}) of the T-matrix become significant, thereby affecting the incident and scattering coefficients, which ultimately influence the magnitude and sign of the optical torques.

Supplementary Note 3: forces and torques on particles under elliptical polarization

Simulations of optical forces and torques on particles under elliptical polarizations are shown in Figs. S9a–S9h and S10a–S10h. Elliptical polarization combines SAM with an asymmetry determined by the degree of ellipticity, resulting in a non-uniform optical field. This asymmetry significantly alters the optical force distribution around particles, leading to shifts in stable rotation angles and changes in the magnitude of optical torque compared to circular polarization. Simulations were conducted using polarization beams with varying degrees of ellipticity (φ=15°,

30°, 60°, 75°). The results indicate that as ellipticity decreases (i.e., the polarization transitions from circular to more elliptical), the magnitude of the optical torque reduces, while the stable rotation angles exhibit notable shifts. Furthermore, the interplay between positive and negative torques becomes increasingly complex, potentially inducing oscillatory rotational dynamics or slower stabilization.

Table S1 Parameter configurations of different prismatic polygons

n (The number of vertices of the polygon.)	(R, h) (nm)	$(\theta_1, \theta_2, \ldots, \theta_n)$ (°)	Corresponding to the numbering in Fig. S1.
3 (Equilateral triangle)	(339.81, 169.90)	(0, 120, 240)	(c)
3 (General triangle)	(356.13, 178.07)	(0, 90, 240)	(i)
4 (Square)	(273.85, 136.93)	(0, 90, 180, 270)	(d)
4 (General quadrilateral)	(291.14, 145.57)	(0, 90, 135, 240)	(j)
5 (Regular pentagon)	(251.17, 125.59)	(0, 72, 144, 216, 288)	(e)
5 (General pentagon)	(272.43, 136.22)	(0, 45, 135, 255, 330)	(k)
6 (Regular hexagon)	(240.28, 120.14)	(0, 60, 120, 180, 240, 300)	(f)
6 (General hexagon)	(260.61, 130.30)	(0, 45, 72, 144, 240, 315)	(l)

Table S2 The vertex coefficient α values of different three-dimensional models

Three-dimensional geometry	α
Sphere	0
Ellipsoid	0
Cylinder (with a square side view)	0.4
Cylinder (with a rectangular side view)	0.4
Regular triangular prism	0.7
General triangle prism	0.7
Regular quadrangular prism	0.8
General quadrilateral prism	0.8
Regular pentagonal prism	0.9
General pentagon prism	0.9
Regular hexagonal prism	1.0
General hexagon prism	1.0

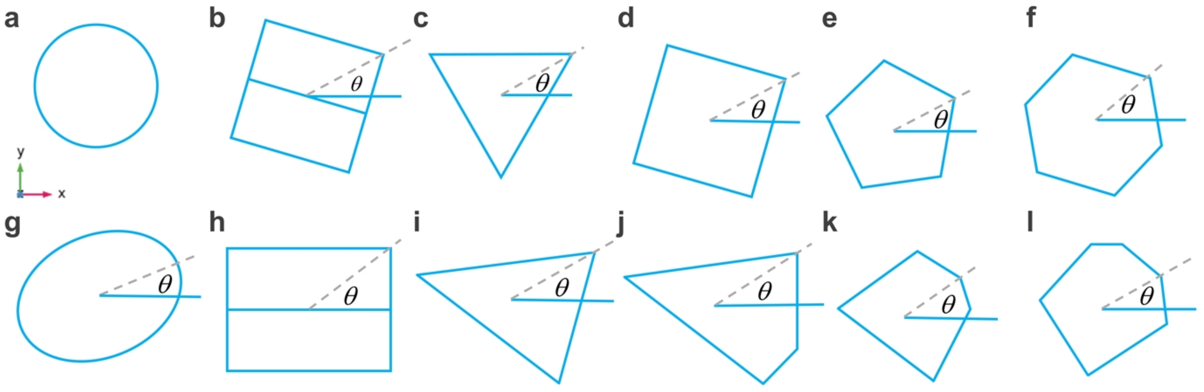

Fig. S1 Projection diagrams of different three-dimensional geometries in the *x*-*y* plane. (**a**) Sphere, (**b**) Cylinder (with a square side view), (**c**) Regular triangular prism, (**d**) Regular quadrangular prism, (**e**) Regular pentagonal prism, (**f**) Regular hexagonal prism, (**g**) Ellipsoid, (**h**) Cylinder (with a rectangular side view), (**i**) General triangle prism, (**j**) General quadrilateral prism, (**k**) Regular pentagonal prism, (**l**) Regular hexagonal prism.

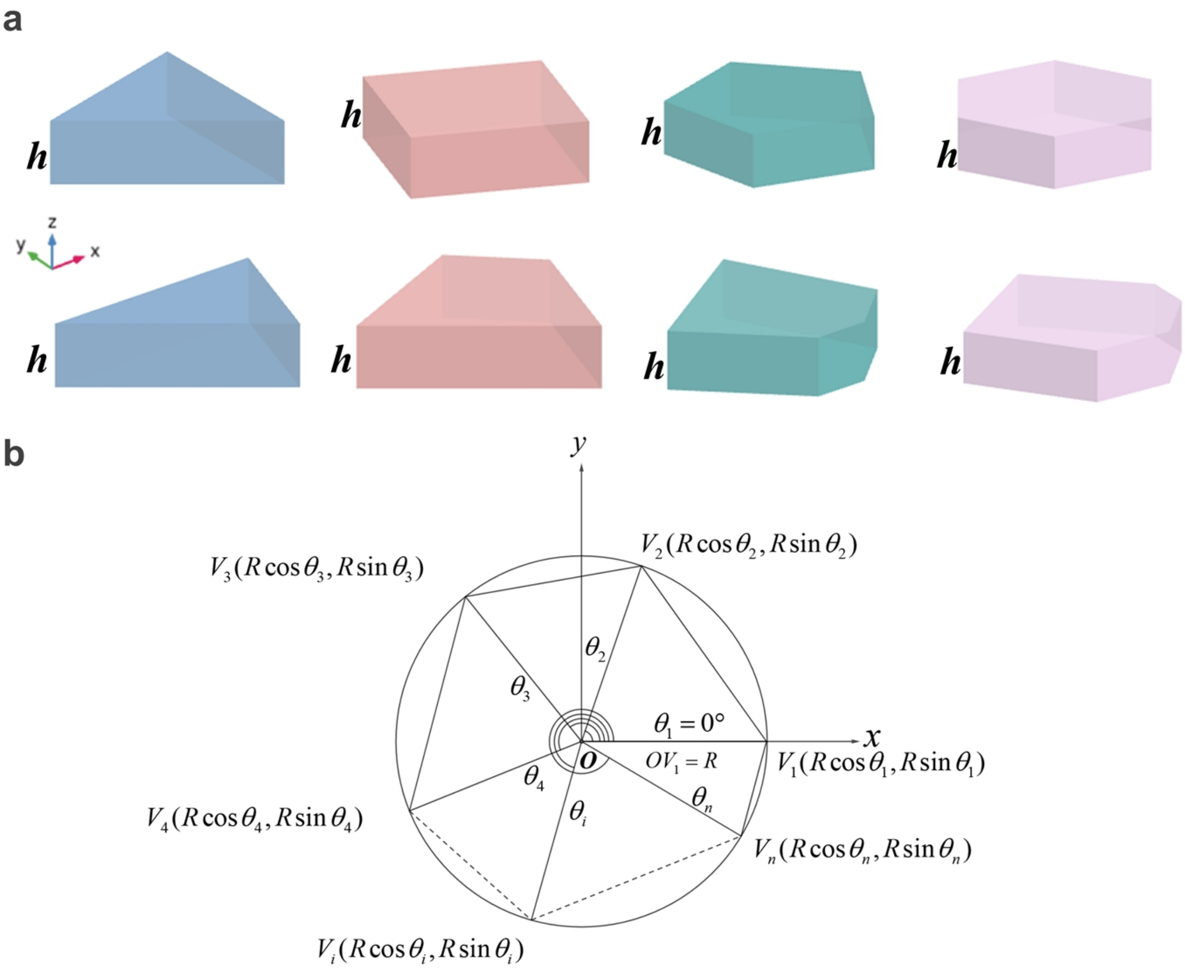

Fig. S2 Three-dimensional diagram of a prism and the construction process of a two-dimensional polygon. (**a**) From left to right are 3D schematic diagrams of triangular, quadrilateral (rectangular), pentagonal, and hexagonal prisms, respectively. Up: Regular prism; Down: General prism; (**b**) Take n points on a circle with a radius of R as the vertices of an n-sided polygon to form the base of the prism**.**

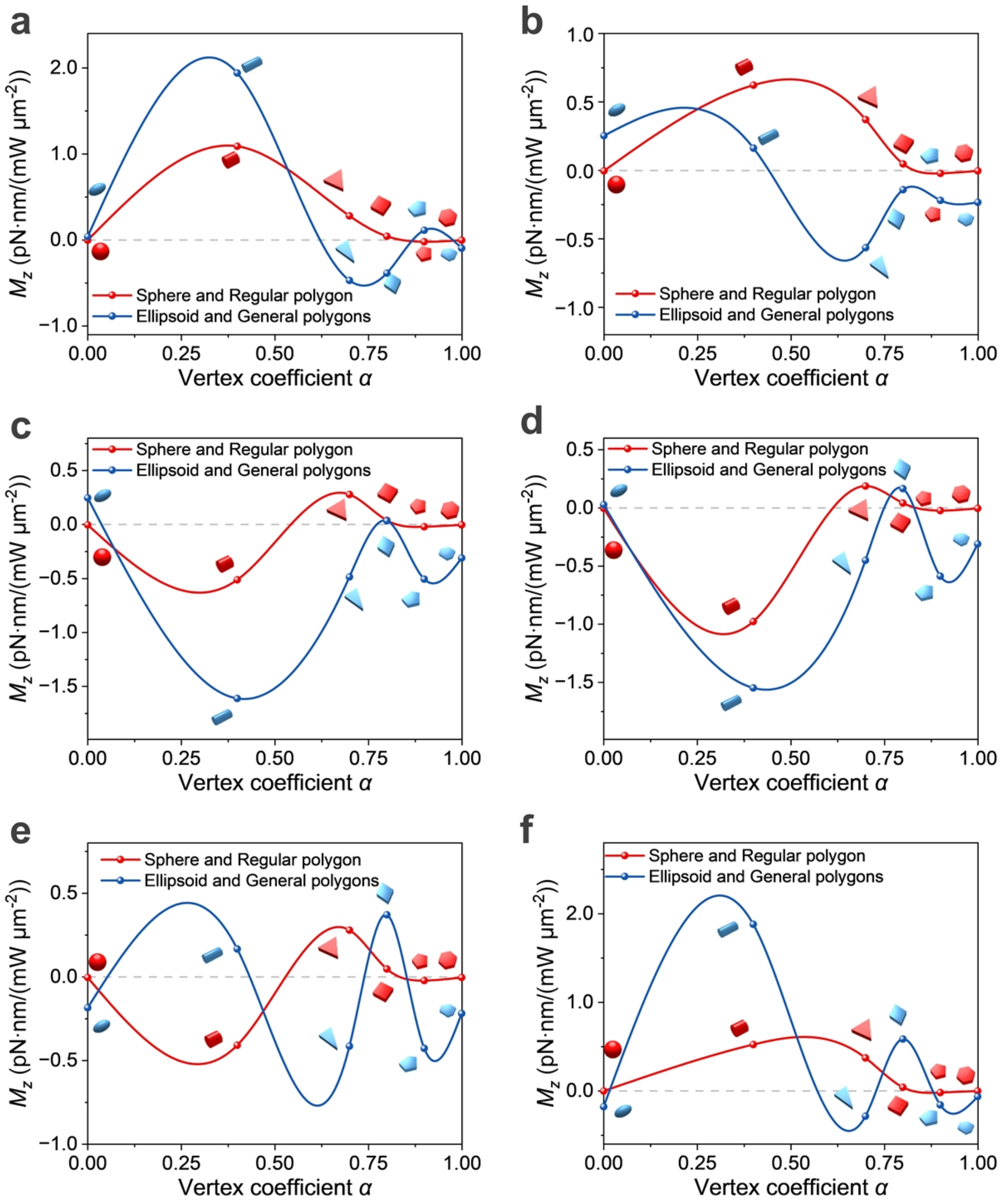

Fig. S3 Simulations of OTs as a function of the vertex coefficient α with different rotation angles (0°−150°). (a)−(f): Simulation of shape evolution to the OTs. The sphere and ellipsoid can be evolved to various shapes by changing the vertex coefficient α. When the sphere and ellipsoid begin to evolve ($\alpha > 0$), the larger the vertex coefficient, OTs for cylinders or prisms are generally smaller. Rotation angles are 0°, 30°, 60°, 90°, 120°, 150° respectively. In **(a)−(f),** w_x = 1000 nm and w_y = 500 μm. OTs are calculated by normalizing the intensity at (x = 0, y = 0) to 1 W μm^{-2}**.**

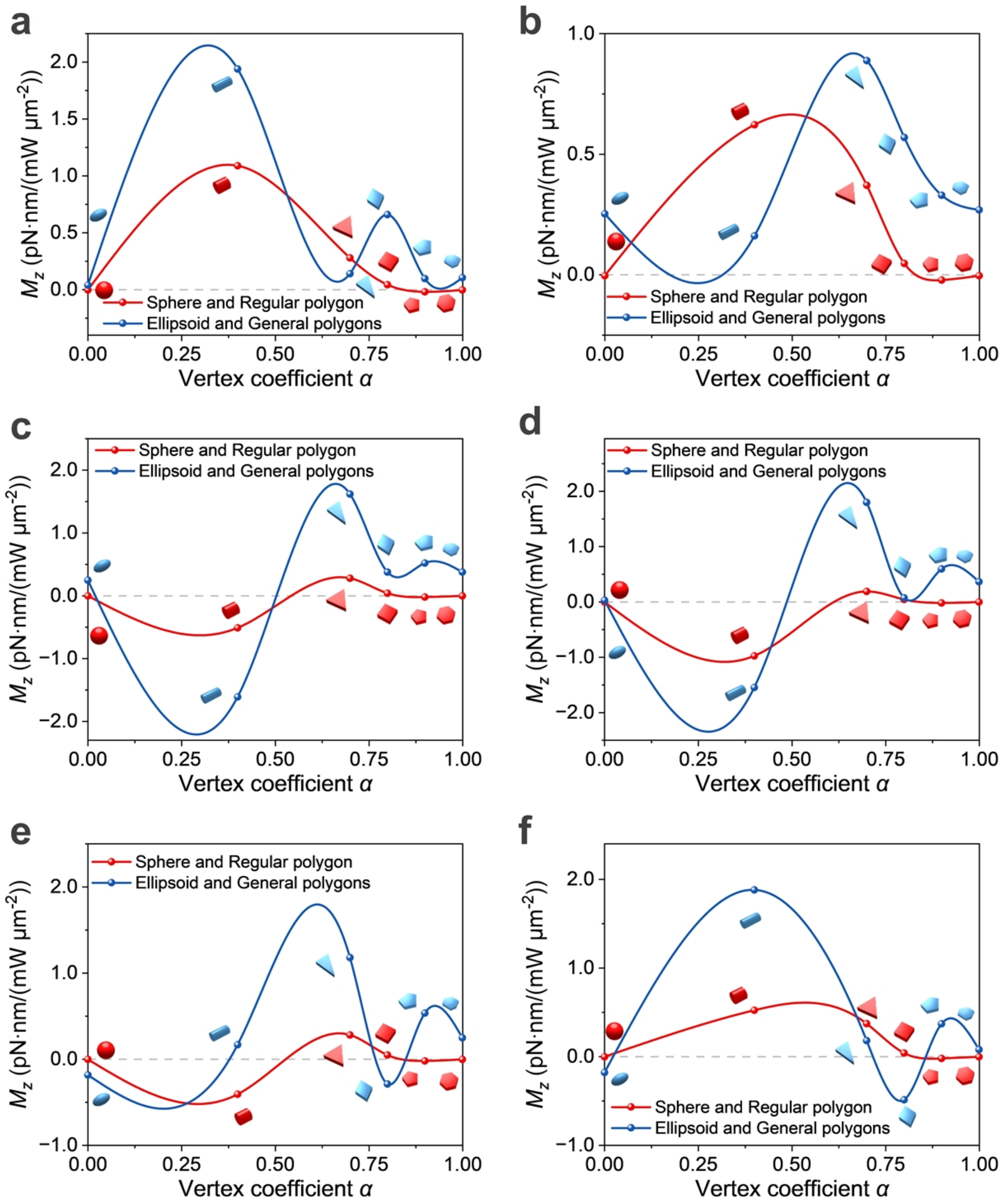

Fig. S4 Simulations of OTs as a function of the vertex coefficient α with different rotation angles (180°−330°). (a)−(f): Simulation of shape evolution to the OTs. The sphere and ellipsoid can be evolved to various shapes by changing the vertex coefficient α. When the sphere and ellipsoid begin to evolve ($\alpha > 0$), the larger the vertex coefficient, OTs for cylinders or prisms are generally smaller. Rotation angles are 180°, 210°, 240°, 270°, 300°, 330° respectively. In **(a)−(f),** $w_x = 1000$ nm and $w_y = 500$ μm. OTs are calculated by normalizing the intensity at ($x = 0, y = 0$) to 1 W μm^{-2}.

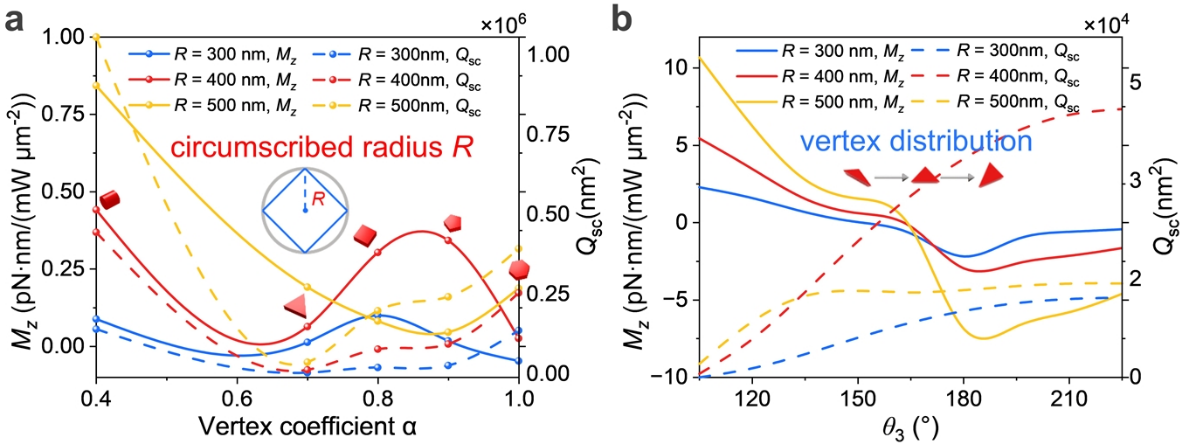

Fig. S5 Simulation of OTs of different *α* or different vertex distributions. (**a**) Optical torques and scattering cross-section curves for the same circumscribed radius (R =300nm, 400nm, 500nm) but different vertex coefficients. (**b**) Optical torques and scattering cross-section curves for the same circumscribed triangular prisms but different vertex distributions. In (**a**) and (**b**), w_x = 1 μm and w_y = 500 μm. OTs are calculated by normalizing the intensity at (x = 0, y = 0) to 1 W μm^{-2}.

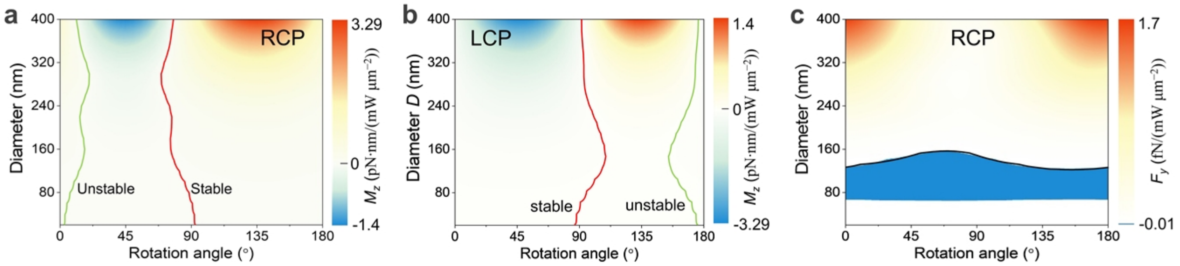

Fig. S6 Simulation of optical torques and forces versus the particle size and rotation angle. (**a**) OT versus the radius and rotation angle of the micro-cylinder (length-diameter ratio: $L/D = 2.5$) when the incident light is RCP(polarization angle $\varphi = 45°$). (**b**) OT versus the radius and rotation angle of the micro-cylinder (length-diameter ratio: $L/D = 1.5$) when the incident light is LCP (polarization angle $\varphi = 135°$). (**c**) The sign of the optical force on the micro-cylinder (length-diameter ratio: $L/D = 1.5$) can reverse with the size and the rotation angle of the particle. The black contour represents zero forces. In (**a**)−(**c**), $w_x = 1$ μm and $w_y = 500$ μm. OTs are calculated by normalizing the intensity at ($x = 0$, $y = 0$) to 1 W μm^{-2}.

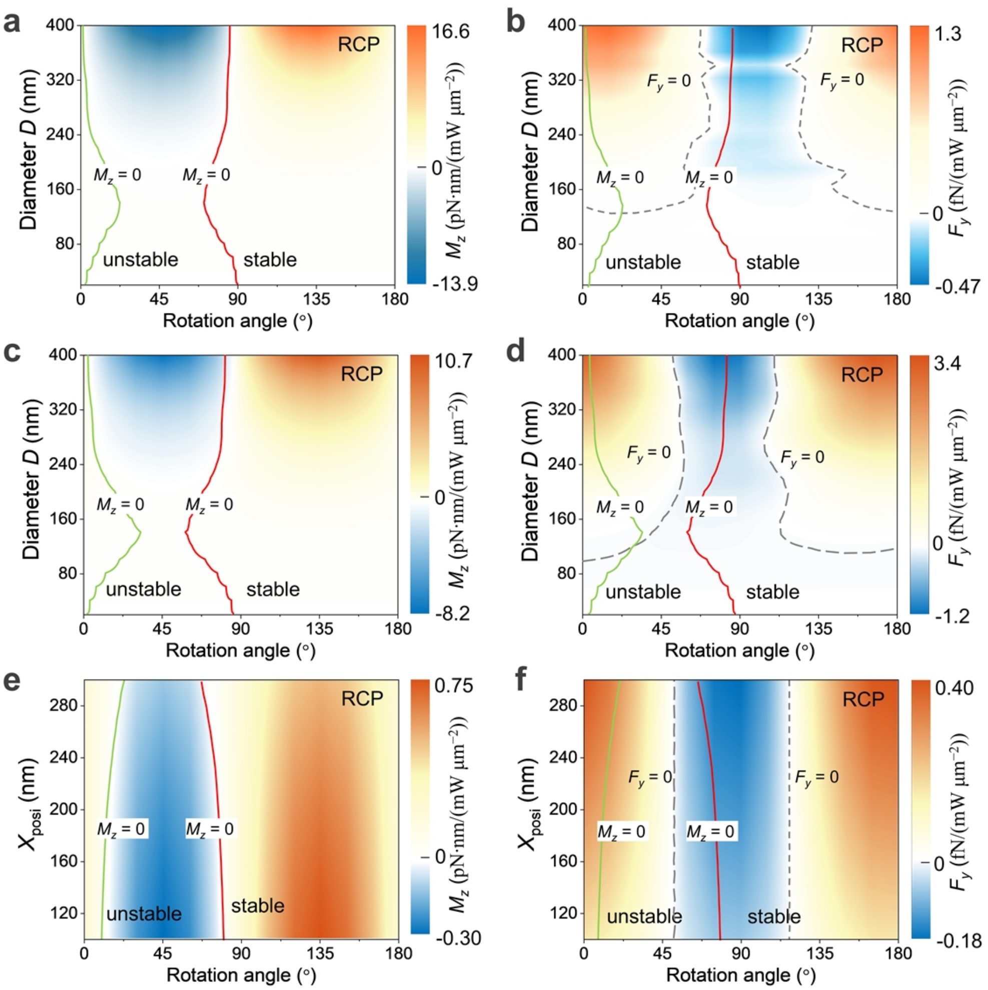

Fig. S7 Simulation of optical torques and forces versus the particle size and rotation angle and the position of the particle on the *x*-axis. Dependence of the (**a**), (**c**)OT and (**b**), (**d**) optical force on the radius and rotation angle (θ) of the micro-cylinder when the incident light is RCP (polarisation angle φ = 45°). The length-diameter ratio of the micro-cylinder is 2.5. Dependence of the (**e**) OT and (**f**) optical force on the position of the particle on the *x*-axis and rotation angle (θ) of the micro-cylinder when the incident light is RCP. (**a**), (**c**), (**e**) Two contours occur in the map, showing stable and unstable equilibrium positions (angles) for positive and negative OTs. (**b**), (**d**), (**f**) Stable angles correspond to negative optical forces, making the micro-cylinder move towards the $-y$ direction. In (**a**) and (**b**), x = 100 nm, in (**c**) and (**d**), x=300 nm, in (**e**) and (**f**), the length-diameter ratio of the micro-cylinder is 2.5 and the diameter of the particle is 200 nm. In (**a**)−(**f**), w_x = 1000 nm and w_y = 500 μm. OTs are calculated by normalising the intensity at (x = 0, y = 0) to 1 W μm^{-2}.

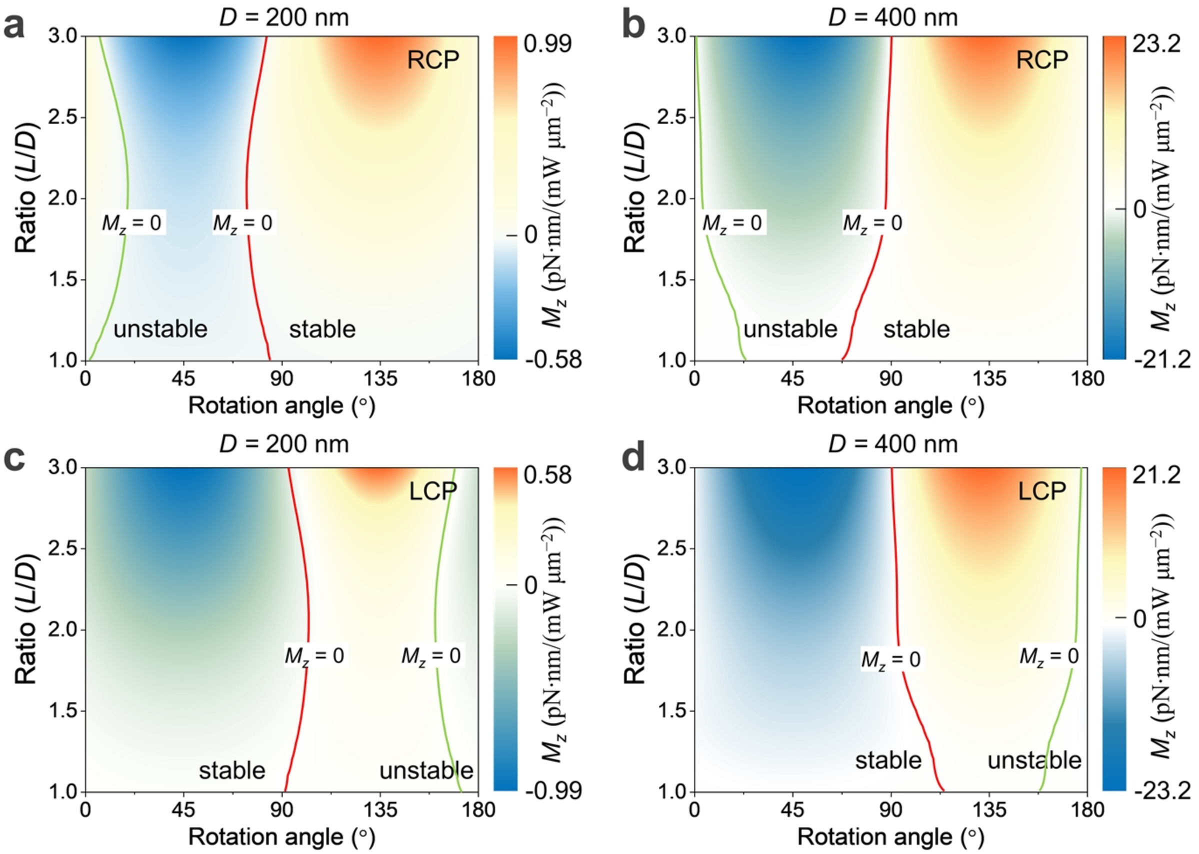

Fig. S8 Simulation of optical torques versus the rotation angle and ratio. (**a**)−(**d**) Dependence of the OTs on the ratio and rotation angle (θ) of the micro-cylinder. In (**a**) and (**b**), the incident light is RCP (polarisation angle φ = 45°), the diameters are 200 nm and 400nm, respectively. In (**c**) and (**d**), the incident light is LCP (polarisation angle φ = 135°), the diameters are 200 nm and 400nm, respectively. In (**a**)−(**d**), w_x = 1 μm and w_y = 500 μm. OTs are calculated by normalizing the intensity at (x = 0, y = 0) to 1 W μm^{-2}.

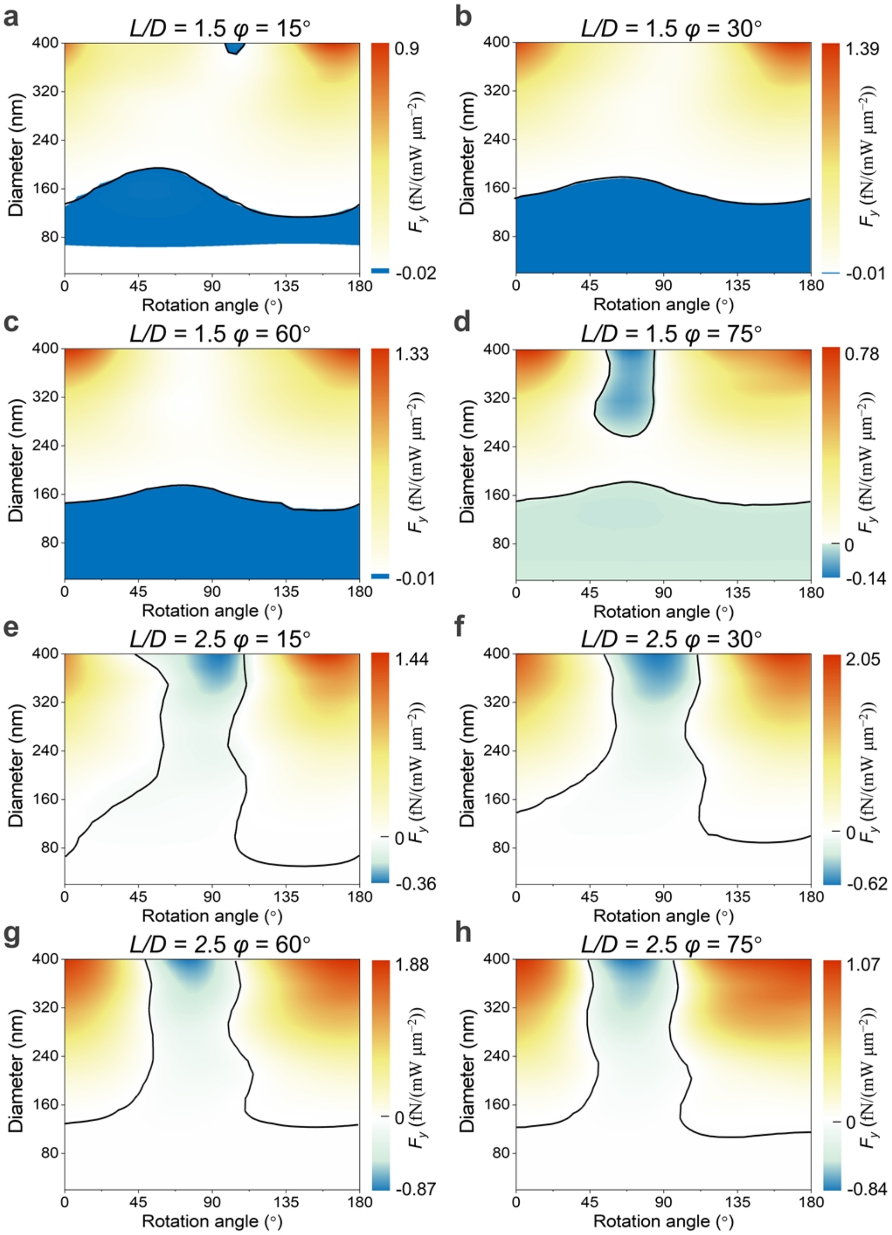

Fig. S9 Simulation of optical forces versus the particle size and rotation angle. (**a**)−(**d**) The sign of the optical force on the micro-cylinder (length-diameter ratio: $L/D = 1.5$) can reverse with the size and the rotation angle of the particle. The black contours represent zero forces. In (**a**)−(**d**), **t**he polarization angles are 15°, 30°, 60°, and 75°, respectively. (**e**)−(**h**) The sign of the optical force on the micro-cylinder (length-diameter ratio: $L/D = 2.5$) can reverse with the size and the rotation angle of the particle. The black contours represent zero forces. In (**a**)−(**h**), $w_x = 1$ μm and $w_y = 500$ μm. OTs are calculated by normalizing the intensity at ($x = 0$, $y = 0$) to 1 W μm^{-2}.

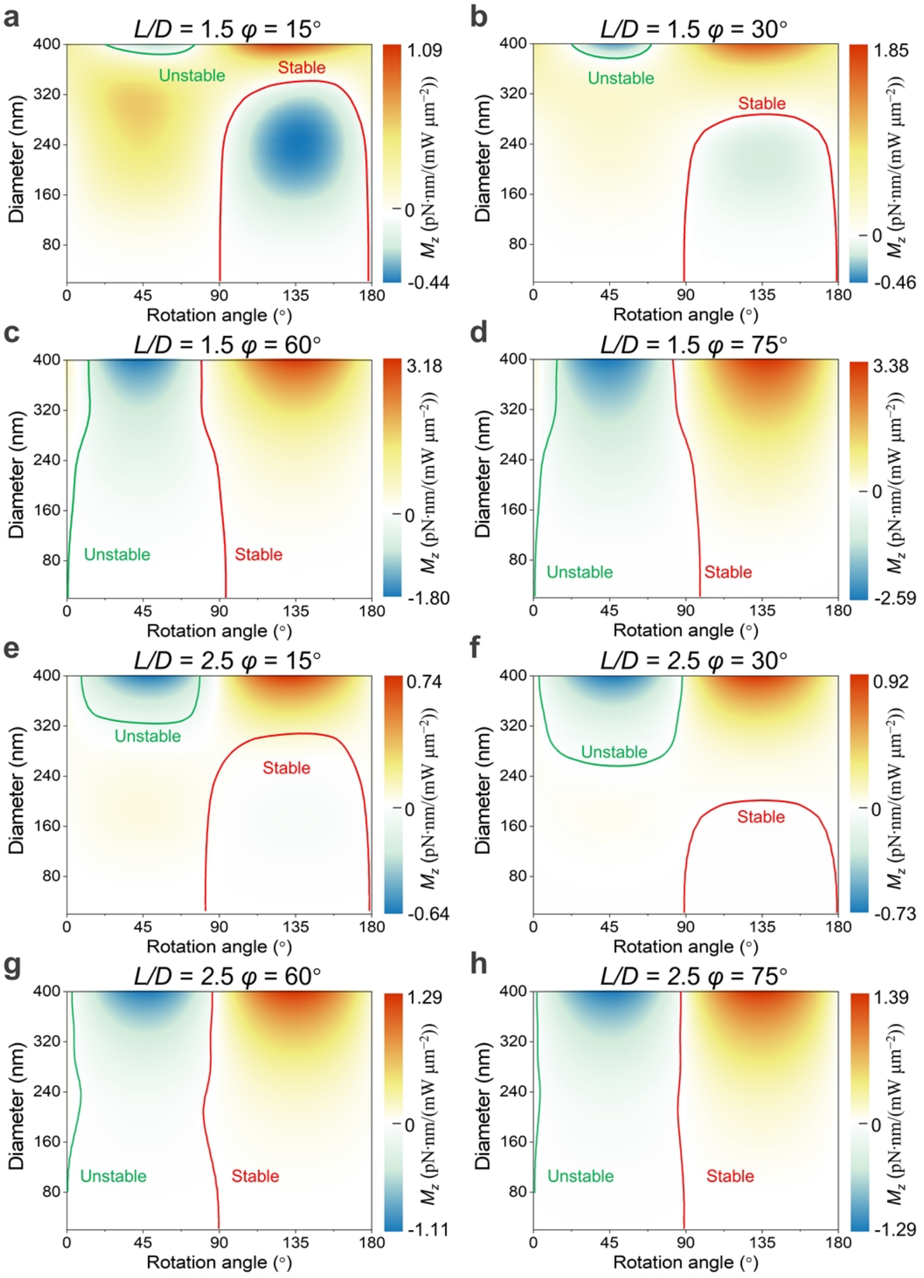

Fig. S10 Simulation of optical torques versus the particle size and rotation angle. (**a**)−(**d**) OT versus the radius and rotation angle of the micro-cylinder (length-diameter ratio: $L/D = 1.5$) when the incident light is elliptically polarized light. In (**a**)−(**d**), polarization angle $\varphi = 15°, 30°, 60°, 75°$, respectively. (**e**)−(**h**) OT versus the radius and rotation angle of the micro-cylinder (length-diameter ratio: $L/D = 2.5$) when the incident light is elliptically polarized light. In (**e**)−(**h**), polarization angle $\varphi = 15°, 30°, 60°, 75°$, respectively. In (**a**)−(**h**), $w_x = 1$ μm and $w_y = 500$ μm. OTs are calculated by normalizing the intensity at ($x = 0$, $y = 0$) to 1 W μm^{-2}.

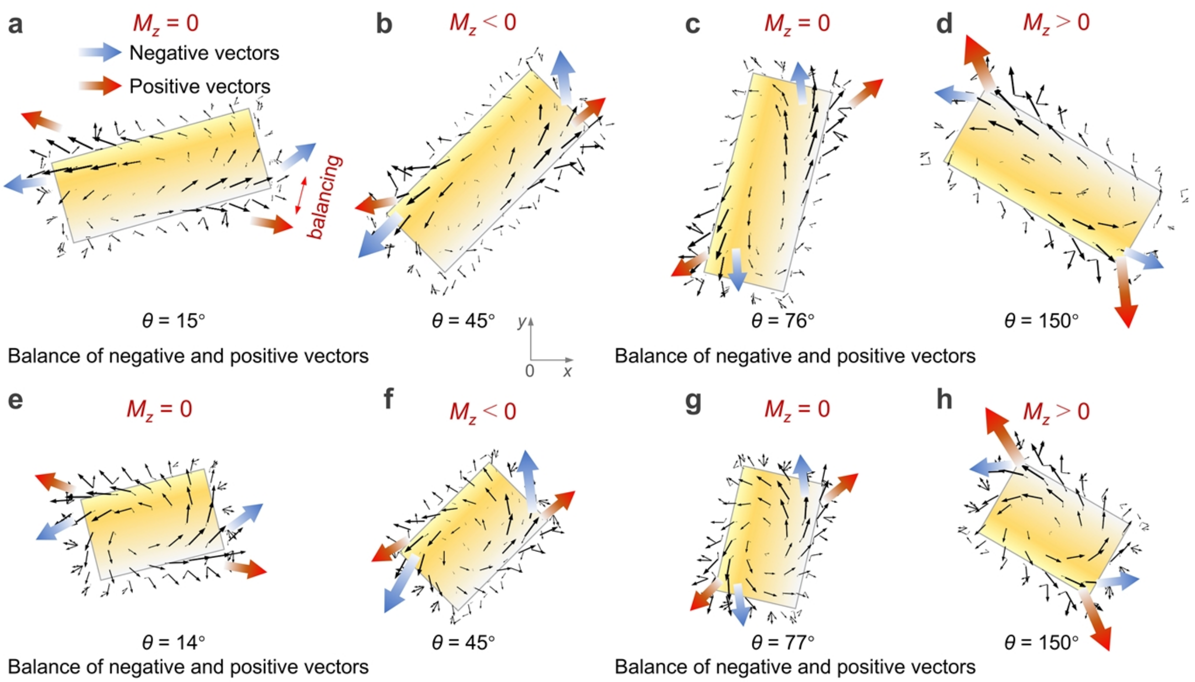

Fig. S11 Plot of force vectors surrounding the micro-cylinder. (**a**)−(**d**) The length-diameter ratio of the micro-cylinder is 2.5. (**e**)–(**h**) The length-diameter ratio of the micro-cylinder is 1.5. (**a**) $\theta = 15°$; (**b**) $\theta = 45°$, (**c**) $\theta = 76°$, (**d**) $\theta = 150°$, (**e**) $\theta = 14°$, (**f**) $\theta = 45°$, (**g**) $\theta = 77°$, (**g**) $\theta = 150°$. Negative and positive vectors denote vectors most likely generating negative and positive OTs, respectively. The OT becomes zero under the balance of negative and positive OTs. When the negative vectors become prominent, the OT is negative, as shown in the upper row of Figs. 2a and S5a. In contrast, the positive OT emerges when the positive vectors become prominent, as shown in the lower row of Figs. 2a and S5a. The diameter D of the micro-cylinder is 200 nm. $w_x = 1000$ nm and $w_y = 500$ μm. OTs are calculated by normalizing the intensity at ($x = 0$, $y = 0$) to 1 W μm^{-2}.

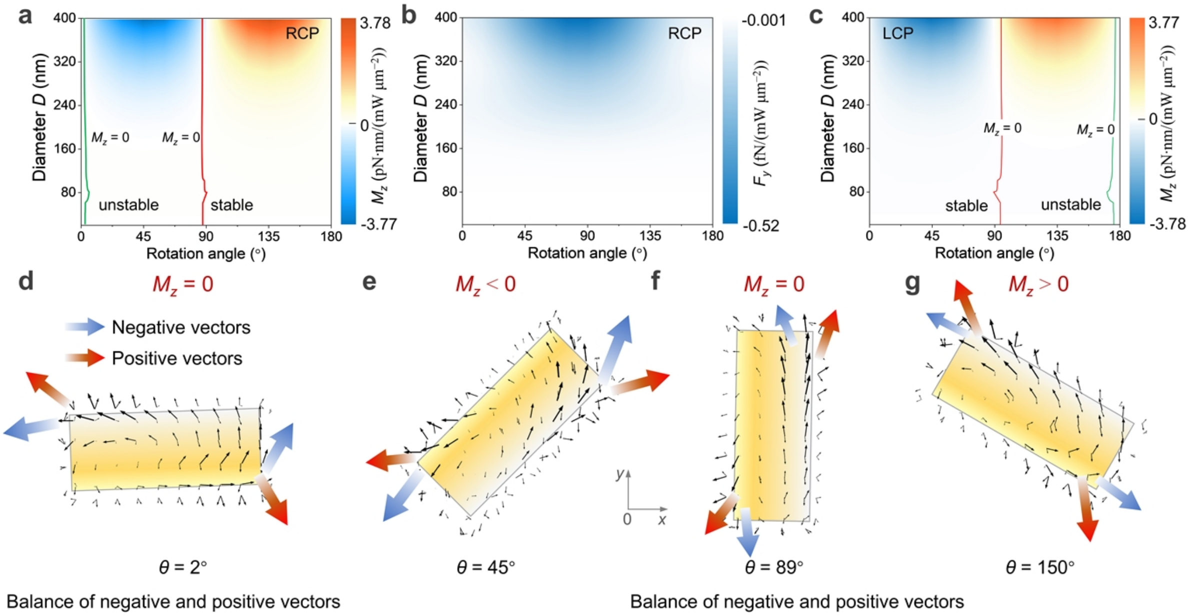

Fig. S12 Simulation of optical torques and forces versus the biological particle size and rotation angle plot of force vectors surrounding the biological micro-cylinder. (**a**) OT versus the radius and rotation angle of the micro-cylinder (length-diameter ratio: L/D = 2.5) when the incident light is RCP (polarization angle φ = 45°). (**b**) The sign of the optical force on the micro-cylinder (length-diameter ratio: L/D = 2.5) can reverse with the size and the rotation angle of the particle. The black contour represents zero forces. (**c**) OT versus the radius and rotation angle of the micro-cylinder (length-diameter ratio: L/D = 2.5) when the incident light is LCP (polarization angle φ = 135°). The refractive index of the bioparticles is set to 1.4. (**d**) θ = 2°, (**e**) θ = 45°, (**f**) θ = 89°, (**g**) θ = 150°.The OT becomes zero under the balance of negative and positive OTs. When the negative vectors become prominent, the OT is negative, as shown in the upper row of (**a**). In contrast, the positive OT emerges when the positive vectors become prominent, as shown in the lower row of (**a**). The diameter D of the micro-cylinder in (**d**)−(**g**) is 200 nm. w_x = 1000 nm and w_y = 500 μm. OTs are calculated by normalizing the intensity at (x = 0, y = 0) to 1 W μm^{-2}."

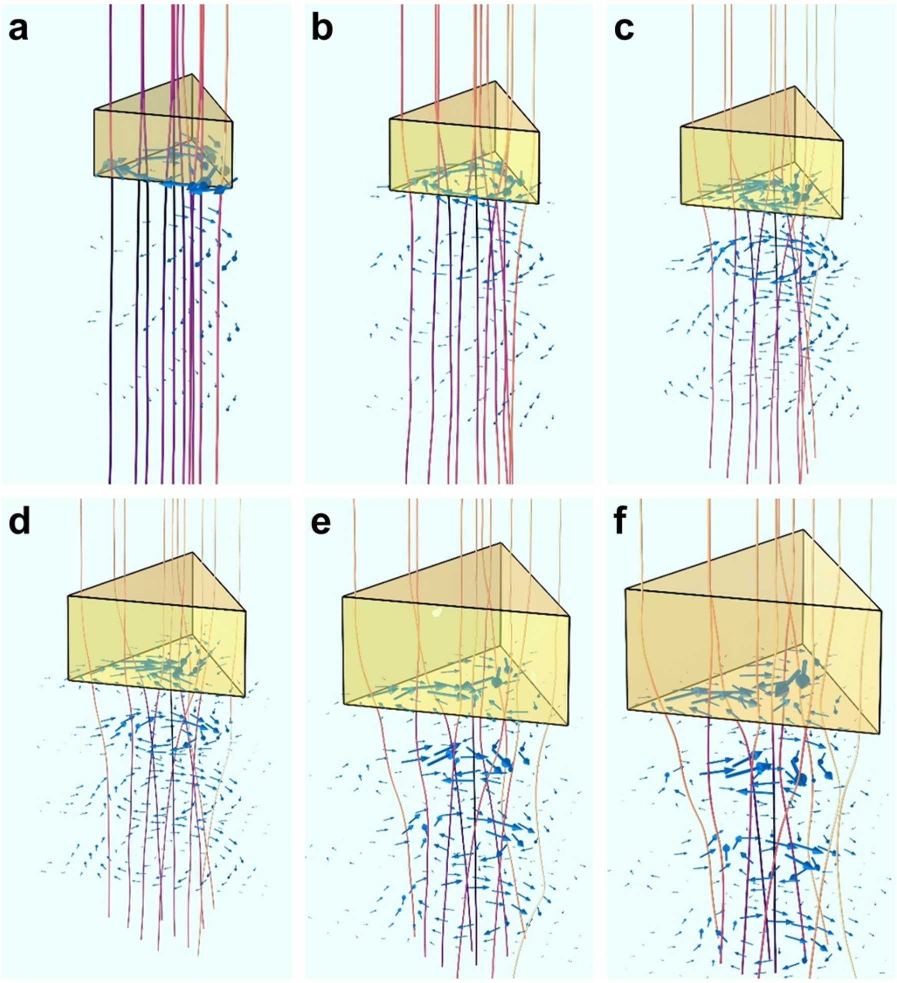

Fig. S13 The energy flow from the top passing through the triangular prism induces vortex-like Poynting vectors on different planes. In (**a**)–(**f**) The side and height of the triangular prism are 200, 400, 600, 800, 1200, 1500 nm and 100, 200, 300, 400, 600, 750 nm, respectively. It can be seen that the nanovortex seems to be a fundamental phenomenon in this design**.**

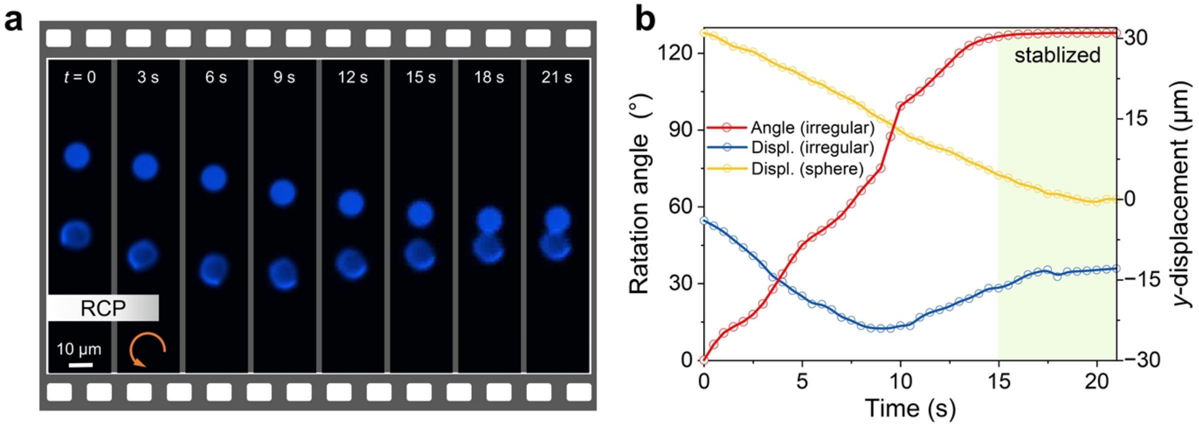

Fig. S14 Experimental observation of the movement of the spherical particle and the dynamic behavior of the irregularly shaped particle. (**a**) Video frames of the movement of the spherical particle and the irregularly shaped particle in the *y* direction, as well as the rotation of the irregularly shaped particle, the incident light is RCP (polarization angle is 45°). Scale bar, 10 μm. (**b**) Retrieved trajectories of the sphere and irregularly shaped particles, as well as rotation angles of the irregularly shaped particle. In (**a**) and (**b**), laser powers are 500 mW.

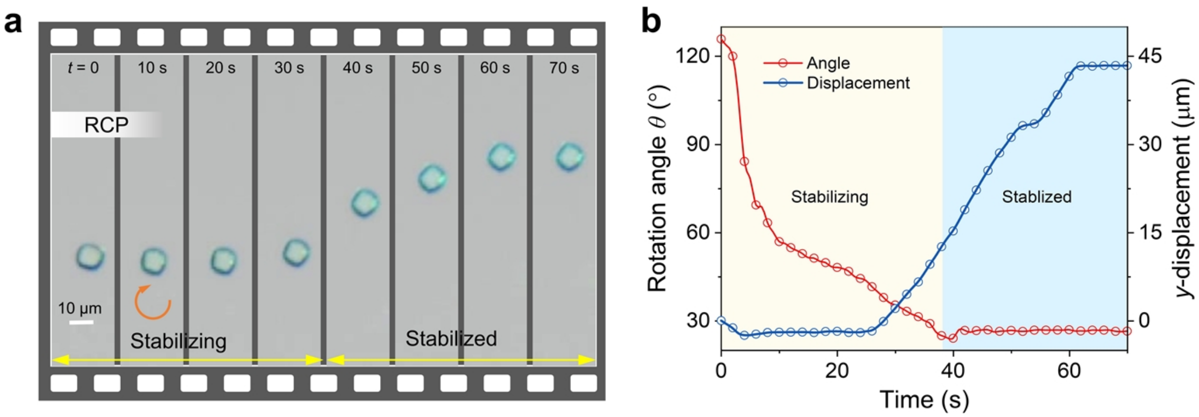

Fig. S15 Experimental observation of the movement and rotation of particles with irregular quadrilateral shape. (**a**) Video frames of the *y*-directional movement and rotation of an irregular quadrilateral particle under the RCP light. Scale bar, 10 μm. (**b**) Retrieved trajectories and rotation angles of the irregular quadrilateral particle. In (**a**) and (**b**), laser powers are 500 mW.

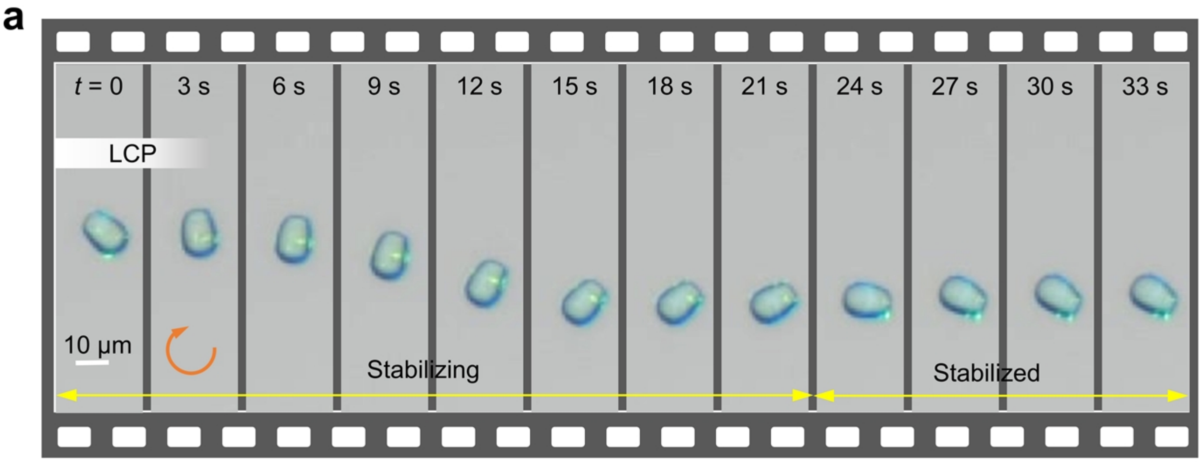

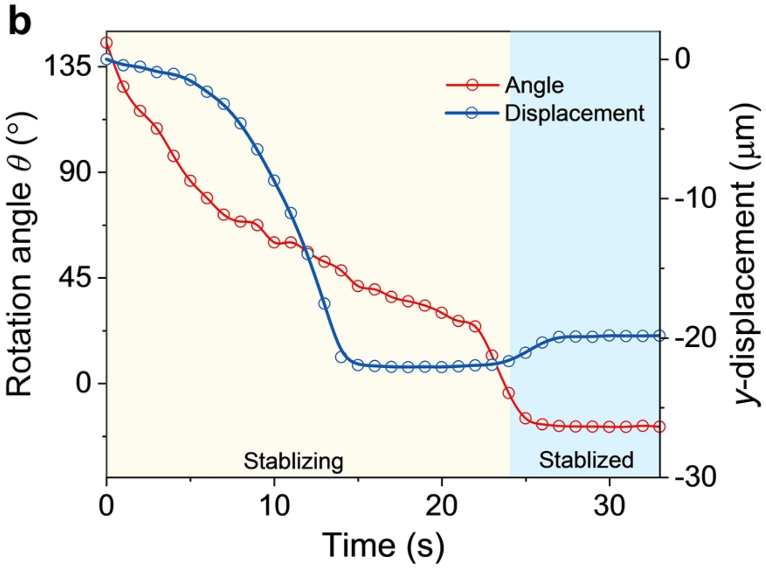

Fig. S16 Experimental observation of the movement and rotation of the ellipsoid-shaped particle (**a**) Video frames of the *y*-directional movement and rotation of an ellipsoid-shaped particle under the LCP light. The particle moves downwards and rotates clockwise. Scale bar, 10 μm. (**b**) Retrieved trajectories and rotation angles of the ellipsoid-shaped particle. In (**a**) and (**b**)**,** laser powers are 500 mW.

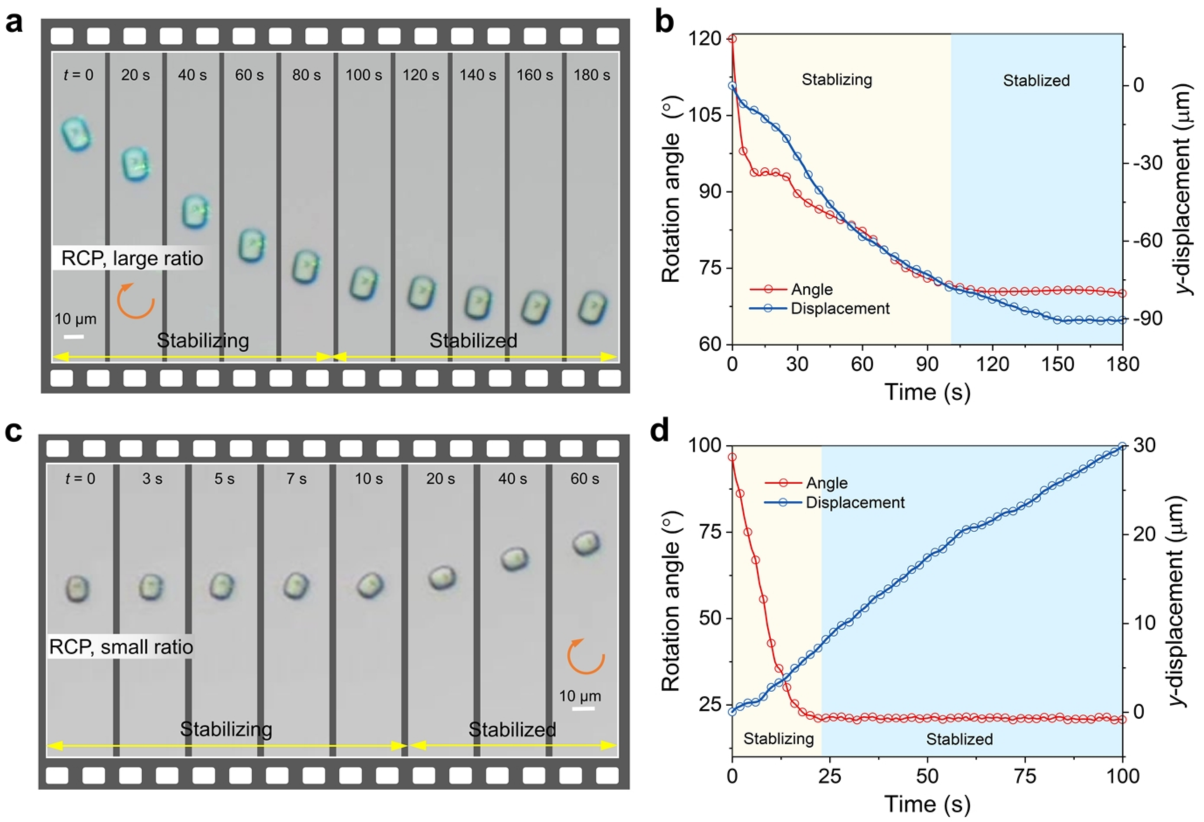

Fig. S17 Experimental observation of the movement and rotation of cylinder particles with different ratios under the right-handed circularly polarized light. (**a**) and (**c**) Video frames of the lateral movement and rotation of different cylinder particles with different ratios under the RCP light. (**a**) Video frame shows that particles with a large ratio move downwards and rotate clockwise, the final angle stabilizes at ~70°. Scale bar, 10 μm. (**b**) Retrieved trajectories and rotation angles of the particle in (**a**). (**c**) Video frame shows that particles with a small ratio move upwards and rotate clockwise, the final angle stabilizes at ~20°. Scale bar, 10 μm. (**d)** Retrieved trajectories and rotation angles of the particle in (**c**)**.** The motion and rotation behaviors of particles with small and large ratios are consistent with the simulation results shown in Figs. 3a and b. In (**a**)−(**d**), laser powers are 500 mW.

References

1. Shi, Y. et al. Inverse Optical Torques on Dielectric Nanoparticles in Elliptically Polarized Light Waves. *Phys. Rev. Lett.* **129**, 053902 (2022).
2. Bliokh, K.Y., Bekshaev, A.Y. & Nori, F. Extraordinary momentum and spin in evanescent waves. *Nat. Commun.* **5**, 3300 (2014).
3. Draine, B. T. The Discrete-Dipole Approximation and Its Application to Interstellar Graphite Grains. *Astrophys. J.* **333**, 848 (1988).
4. Bohren, C. F. & Huffman, D. R. Absorption and Scattering of Light by Small Particles. (1998)
5. Yunos, N. M. *et al.* The depolarization factors for ellipsoids and some of their properties. *Mal. J. Fund. Appl. Sci.* **15**, 784-789 (2019).
6. Xu, X. et al. Gradient and curl optical torques. *Nat. Commun.* **15**, 6230 (2024).
7. Simpson, S. H. & Hanna, S. Optical trapping of spheroidal particles in Gaussian beams. *JOSA A* **24**, 430-443 (2007).
8. Travis, L., Mishchenko, M. I. & Lacis, A. A. Scattering, Absorption, and Emission of Light by Small Particles (Cambridge university press, 2002)
9. Wang, S.B. & Chan, C.T. Lateral optical force on chiral particles near a surface. *Nat. Commun.* **5**, 3307 (2014).
